# Saprophytic and pathogenic fungi in the Ceratocystidaceae differ in their ability to metabolize plant-derived sucrose

**DOI:** 10.1186/s12862-015-0550-7

**Published:** 2015-12-07

**Authors:** M. A. Van der Nest, E. T. Steenkamp, A. R. McTaggart, C. Trollip, T. Godlonton, E. Sauerman, D. Roodt, K. Naidoo, M. P. A. Coetzee, P. M. Wilken, M. J. Wingfield, B. D. Wingfield

**Affiliations:** Department of Genetics, Forestry and Agricultural Biotechnology Institute (FABI), University of Pretoria, Private Bag X20, Pretoria, 0028 South Africa; Department of Microbiology and Plant Pathology, Forestry and Agricultural Biotechnology Institute (FABI), University of Pretoria, Private Bag X20, Pretoria, 0028 South Africa

**Keywords:** *Ceratocystis*, *Huntiella*, Vacuolar invertases, Cell wall invertases, Sucrolytic ability, Gene family evolution, Molecular dating, Transposable elements, Paralog

## Abstract

**Background:**

Proteins in the Glycoside Hydrolase family 32 (GH32) are carbohydrate-active enzymes known as invertases that hydrolyse the glycosidic bonds of complex saccharides. Fungi rely on these enzymes to gain access to and utilize plant-derived sucrose. In fungi, GH32 invertase genes are found in higher copy numbers in the genomes of pathogens when compared to closely related saprophytes, suggesting an association between invertases and ecological strategy. The aim of this study was to investigate the distribution and evolution of GH32 invertases in the Ceratocystidaceae using a comparative genomics approach. This fungal family provides an interesting model to study the evolution of these genes, because it includes economically important pathogenic species such as *Ceratocystis fimbriata*, *C. manginecans* and *C. albifundus*, as well as saprophytic species such as *Huntiella moniliformis*, *H. omanensis* and *H. savannae*.

**Results:**

The publicly available Ceratocystidaceae genome sequences, as well as the *H. savannae* genome sequenced here, allowed for the identification of novel GH32-like sequences. The *de novo* assembly of the *H. savannae* draft genome consisted of 28.54 megabases that coded for 7 687 putative genes of which one represented a GH32 family member. The number of GH32 gene family members appeared to be related to the ecological adaptations of these fungi. The pathogenic *Ceratocystis* species all contained two GH32 family genes (a putative cell wall and a putative vacuolar invertase), while the saprophytic *Huntiella* species had only one of these genes (a putative cell wall invertase). Further analysis showed that the evolution of the GH32 gene family in the Ceratocystidaceae involved transposable element-based retro-transposition and translocation. As an example, the activity of a *Fot5*-like element likely facilitated the assembly of the genomic regions harbouring the GH32 family genes in *Ceratocystis*.

**Conclusions:**

This study provides insight into the evolutionary history of the GH32 gene family in Ceratocystidaceae. Our findings suggest that transposable elements shaped the evolution of the GH32 gene family, which in turn determines the sucrolytic activities and related ecological strategies of the Ceratocystidaceae species that harbour them. The study also provides insights into the role of carbohydrate-active enzymes in plant-fungal interactions and adds to our understanding of the evolution of these enzymes and their role in the life style of these fungi.

**Electronic supplementary material:**

The online version of this article (doi:10.1186/s12862-015-0550-7) contains supplementary material, which is available to authorized users.

## Background

Glycoside hydrolases (GHs; often referred to as glycosidases or carbohydrases) that target the terminal β-(2 → 1) fructosidic bonds found in sucrose and various oligo- and polysaccharides (e.g., fructans, inulin and levan) are functionally designated as invertases [1–3]. These enzymes are classified by their pH optima into the so-called neutral/alkaline invertases that belong to GH family 100 (GH100) and the acid invertases that belong to GH family 32 (GH32; [4]). While GH100 invertases are closely related to the cyanobacterial invertases, the GH32 invertases are closely related to invertases of respiratory eukaryotes such as yeasts and aerobic bacteria such as *Bacillus* [5]. Like the GH100 family, proteins in the GH32 family have a range of activities [6]. Those specific to GH32 include enzymes with β-fructofuranosidase (EC 3.2.1.26), inulinase (EC 3.2.1.7, EC 3.2.1.64, EC 3.2.1.80), levanase (EC 3.2.1.65), fructosyltransferase (EC 2.4.1.99, EC 2.4.1.100) and fructosidase (EC 3.2.1.153, EC 3.2.1.154) activities [2, 6].

At the structural level, GH32 together with GH43, GH62 and GH68, are classified as members of the furanosidase (or β-fructosidase) superfamily [7, 8]. These four GH families have a five-blade β-propeller catalytic domain in common, but differ in their mechanisms for glycosidic bond hydrolysis [7]. Those in GH32 and GH68 (designated as clan GH-J) cleave glycosidic bonds in a retaining manner (i.e., retaining of the substrate anomeric configuration), while those in GH43 and GH62 (designated clan GH-F) cleave glycosidic bonds in an inverting manner (i.e., inversion of the substrate anomeric configuration) [8]. GH32 enzymes differ from GH68 in that they contain an additional C-terminal β-sheet domain that probably allows for the maintenance of structural stability during protein oligomerisation [9]. In terms of their known distribution across the Tree of Life, GH32 and GH43 occur in plants, fungi and bacteria, GH68 in bacteria only and GH62 in bacteria and fungi [10].

GH32 enzymes have diverse biological roles and they are also exploited for commercial and medical purposes. In plants they influence developmental processes, supply carbohydrates to sink tissues and link intracellular and extracellular stimuli to regulate source/sink relations [11, 12]. In bacteria and fungi they allow for the utilization of plant-derived sucrose as a carbon source [2, 13]. From an industrial perspective, microbial GH32 invertases have various applications [14]. They are used in the confectionery industry to produce short-chain fructooligosaccharides (FOS), which are utilized as calorie-free and non-cariogenic sweeteners [1]. These enzymes are also associated with benefits for human health, for example as immune boosters and antioxidants [15].

Fungi utilize plant-derived sucrose through the production of different GH32 enzymes [2, 16]. In *Saccharomyces cerevisiae*, two forms of this protein are produced. The first is a non-glycosylated cytoplasmic form that is constitutively expressed, while the second is a glycosylated form that is secreted and repressed by the presence of glucose in the growth medium [17]. Indeed, the overall access to plant-synthesized sucrose appears to be determined by the GH32 family gene copy number [2]. It was previously shown that the number of GH32 genes in a particular species is related to its ecological strategy [2, 13]. Plant pathogens typically show GH32 family expansions, likely because these enzymes play a key role in pathogen nutrition [2, 18]. In contrast, sucrose-independent species, such as animal pathogens and some mycorrhizal fungi, generally lack the genes encoding these enzymes [2]. Such differences in gene copy number can arise from intrinsic molecular processes like unequal crossover and chromosomal duplication, or from processes linked to the activity of mobile genetic elements like transposons [19].

The potential link between GH32 protein family evolution and ecological adaptation has not been explored in the Ceratocystidaceae. This monophyletic family of fungi includes several ecologically diverse lineages that lend themselves to functional comparison [20]. The genus *Huntiella*, for example, includes exclusively saprophytic species that typically colonize the wounded tissues of trees and other plants [20]. In contrast, the economically important genus *Ceratocystis* includes mainly pathogens of woody and herbaceous plants, some of which cause devastating tree diseases [21, 22]. Notable examples include the sweet potato pathogen *C. fimbriata* [23], the mango pathogen *C. manginecans* [24]*,* and the *Acacia* pathogen *C. albifundus* [25]. Despite the availability of whole genome sequence information for all three of the latter species, as well as for *H. moniliformis* [21] and *H. omanensis* [26, 27], very little is known regarding their GH32 genes, much less their overall sucrolytic capabilities. In this regard, only one GH32 gene and its associated product has been characterised (i.e., *CmINV* of *H. moniliformis*) and tested for its ability to produce FOS [28].

This study considered the structure and evolution of the GH32 protein family in pathogenic and non-pathogenic species in the Ceratocystidaceae. The specific research objectives were: (*i*) Sequence and assemble the genome of a third *Huntiella* species, *H. savannae*, to allow for meaningful genomic comparison between *Huntiella* and *Ceratocystis*; (*ii*) Identify and annotate putative GH32 family genes in *H. savannae* and publicly available genomes of *Ceratocystis* and *Huntiella* using an *in silico* approach; (*iii*) Infer the evolutionary history of the GH32 family in Ceratocystidaceae and other Sordariomycetes; (iv) Identify potential genomic processes that shaped the evolution of the GH32 gene family.

## Methods

### Genome sequences

Genome sequence information for three *Huntiella* species and three *Ceratocystis* species was utilized in this study (Table 1). Genomes for *H. moniliformis* ([GenBank:JMSH00000000]; [26]), *H. omanensis* ([GenBank:SUI00000000]; [27]), *C. manginecans* ([GenBank:JJRZ01000000]; [26]), *C. fimbriata* ([GenBank:APWK00000000]; [21]) and *C. albifundus* ([GenBank:JSSU00000000]; [27]) were generated in previous studies and are available from the GenBank database of the National Centre for Biotechnology Information (NCBI; http://www.ncbi.nlm.nih.gov/). The genome sequence for *H. savannae* (isolate CMW17300, [29]) was determined in the current study (see below).

**Table 1:** Genome information of the *Huntiella*, *Ceratocystis* and Sordariomycetes species included in this study

Taxon	Assembly size (Mbp)	Number of scaffolds^a^	Number of gene models^b^	References^c^
*C. manginecans*	31.71	980	7 494	Van der Nest et al. 2014.
*C. fimbriata*	29.40	2 641	7 266	Wilken et al. 2013
*C. albifundus*	27.15	939	6 967	Van der Nest et al. 2014.
*H. moniliformis*	25.43	365	6 832	Van der Nest et al. 2014.
*H. savannae*	28.54	361	7 361	This study
*H. omanensis*	31.50	1 638	8 395	Van der Nest et al. 2014.
*Acremonium alcalophilum* v2.0	54.42	13	9 521	JGI
*Anthostoma avocetta* NRRL 3190 v1.0	56.23	786	15 755	JGI
*Apiospora montagnei* NRRL 25634 v1.0	47.67	686	16 992	JGI
*Beauveria bassiana* ARSEF 2860	33.69	235	10 364	Xiao et al. 2012 Scientific Reports 2
*Chaetomium globosum* v1.0	34.90	37	11 124	JGI
*Colletotrichum graminicola* M1.001	51.60	653	12 006	O’Connell et al. 2012 Nat Genet 2012 44:1060–5
*Colletotrichum higginsianum* IMI 349063	49.08	10 235	16 172	O’Connell et al. 2012 Nat Genet 2012 44:1060–5
*Coniochaeta ligniaria* NRRL30616 V.1.0	42.38	135	13 657	JGI
*Cordyceps militaris* CM01	32.27	32	9 651	Zheng et al. 2011 Genome Biol 12:R116
*Cryphonectria parasitica* EP155 v2.0	43.90	26	11 609	JGI
*Daldinia eschscholzii* EC12 v1.0	37.55	398	11 173	JGI
*Eutypa lata* UCREL1	54.01	2 334	11 685	Blanco-Ulate et al. 2013 Genome Announc 1:e00390–13
*Fusarium fujikuroi* IMI 58289	43.83	12	14 813	Wiemann et al. 2013 PLoS Pathog 9:e1003475
*Fusarium graminearum* v1.0	36.45	31	13 322	Cuomo et al. 2007 Science 317:1400–2
*Fusarium oxysporum v1.0*	61.36	114	17708	JGI
*Fusarium verticillioides 7600* v1.0	41.78	36	14 188	JGI
*Nectria haematococca v2.0*	51.15	72	15 707	JGI
*Glomerella acutata v1.0*	50.04	307	15 777	JGI
*Glomerella cingulata 23 v1.0*	58.84	119	18 975	JGI
*Grosmannia clavigera* kw1407	29.79	289	8 312	DiGuistini et al. 2011 PNAS 108:2504–9
*Hypoxylon* sp. CI-4A v1.0	37.70	899	11 712	JGI
*Ilyonectria* sp. v1.0	63.66	325	22 250	JGI
*Metarhizium acridum* CQMa 102	39.42	241	9 849	Gao et al. 2011 PLoS Genet 7:e1001264
*Metarhizium robertsii* ARSEF 23	39.15	176	10 583	Gao et al. 2011 PLoS Genet 7:e1001264
*Myceliophthora thermophila* v2.0	38.74	7	9 110	Berka et al. 2011 Nature Biotech 29:922–927
*Neurospora crassa* OR74A v2.0	41.04	20	10785	JGI
*Neurospora discreta* FGSC 8579 mat A	176.0	37.3	9 948	JGI
*Neurospora tetrasperma* FGSC 2508 mat A v2.0	39.10	81	10 380	Ellison et al. 2011 Genetics 189:55–69
Ophiostoma piceae UAMH 11346	32.84	45	8 884	Haridas et al. 2013 BMC Genomics 14:373
*Phaeoacremonium aleophilum* UCRPA7	47.47	624	8 834	Blanco-Ulate et al. 2013 Genome Announc 1, e00390–13
*Podospora anserina* S mat+	35.01	7	10588	JGI
*Sodiomyces alkalinus* v1.0	43.45	29	9 411	JGI
*Thielavia antarctica* CBS 123565 v1.0	40.66	153	9 204	JGI
*Thielavia appendiculata* CBS 731.68 v1.0	32.74	109	11 942	JGI
*Thielavia arenaria* CBS 508.74 v1.0	30.99	69	10 954	JGI
*Thielavia hyrcaniae* CBS 757.83 v1.0	31.18	251	11 338	JGI
*Thielavia terrestris* v2.0	36.91	6.00	9 813	Berka et al. 2011 Nature Biotech 29:922–927
*Trichoderma atroviride* V2.0	36.10	29.00	11 863	JGI
*Trichoderma asperellum* CBS 433.97 v1.0	40.87	2 282	13 932	JGI
*Trichoderma harzianum* CBS 226.95 v1.0	40.98	532	14 095	JGI
*Trichoderma longibrachiatum* ATCC 18648 v3.0	40.87	2 282	13 932	JGI
*Trichoderma virens* Gv29-8 v2.0	39.00	93	12 427	JGI
*Trichoderma reesei* v2.0	34.10	89	9 129	JGI
*Verticillium dahliae* v1.0	33.83	52	10 535	Klosterman et al. 2011 PLoS Pathogens 7: e1002137

^a^ Number of scaffolds/supercontigs that make up an individual genome assembly

^b^ Number of the genes predicted for each genome assembly

^c^ JGI = Joint Genome Institute portal (www.jgi.doe.gov)

Isolate CMW17300 of *H. savannae* was grown on medium containing 20 g/L malt extract agar (MEA, Biolab, Johannesburg, South Africa). Mycelia were scraped from the growth medium and genomic DNA extracted using a phenol/chloroform protocol as previously described by Barnes et al. [30]. The DNA was then sequenced using the Genome Analyzer IIx platform (Illumina) at the Genome Centre, UC Davis, California, USA. Paired-end libraries with an insert size of approximately 350 and 600 bases were used to produce reads with an average length of 100 bases. CLC Genomics Workbench 6.0.1 (CLC Bio, Aarhus, Denmark) was used to discard poor-quality reads and/or terminal nucleotides at a threshold of Q13 (*P* = 0.05) after which *de novo* assembly was done using Velvet [31], and an optimal K mer length of 67 determined using VelvetOptimiser (http://bioinformatics.net.au/software.velvetoptimiser.shtml). The pre-assemblies were scaffolded using SSPACE v.2.0 [31] with default parameters, except -x = 0 and -k = 20. The gaps were reduced with GapFiller v.2.2.1 [32] using default parameters. Open reading frames (ORFs) were predicted using AUGUSTUS [32] based on the gene models for *Fusarium graminearum* (http://bioinf.uni-greifswald.de/augustus), while genome completeness was evaluated using the Core Eukaryotic Genes Mapping Approach (CEGMA) pipeline [33].

### GH32 gene identification and characterisation

To identify putative GH32 homologs in the genomes considered in this study, we utilized representative sequences that spanned the fungal GH32 gene family phylogeny [2]. These included *Aspergillus oryzae* (XP001823245, Group 1), *A. niger* (ABB59682.1, Group 2), *F. verticillioides* (FVEG10082.3, Group 3), *Botryotinia fuckeliana* (BCIG16010.1, Group 4), *Stagonospora nodorum* (SNOG01192.1, Group 5), *Neurospora crassa* (EAA32020 Group 6), *A. niger* (ABB59678.1 Group 7), *H. moniliformis* (AGV22100.1 Group 8) [29], and *A. terreus* (XP001218601 Group 9). In the various *Huntiella* and *Ceratocystis* genomes, putative invertase homologs were identified by performing local BLAST searches (tblastn, expect (E)-values < 10^−5^) using BioEdit v 7.2.5 [34]. For comparative purposes, putative invertase homologs among representative Sordariomycetes were identified and obtained using BLAST searches (blastp and tblastn, E-values < 10^−5^) on the Joint Genome Institute (JGI) portal (www.genome.jgi.doe.gov) (Table 1).

For the identified genes, functional domains and features of the predicted proteins were annotated using InterProScan (v.4.8) (http://www.ebi.ac.uk/Tools/pfa/iprscan/), NCBI’s Conserved Domain (CD) (http://www.ncbi.nlm.nih.gov/Structure/cdd/wrpsb.cgi) and Pfam searches (http://pfam.xfam.org/search), as well as SignalP v.4.1 (www.cbs.dtu.dk/services/SignalP/) and NetNGlyc v.1.0 (www.cbs.dtu.dk/services/NetNGlyc/) analyses. Sub-cellular localization analysis was performed using SignalP. Three-dimensional (3D) models of the N-terminal and C-terminal domains were respectively generated and visualised using the Swiss-Model Web server (http://www.expasy.org/swissmod/SWISS-MODEL.html) and Swiss-PdbViewer v.4.04 (http://spdbv.vital-it.ch/). To predict the 3D structure of the identified invertases, a 3D structure of a fructosyltransferase in *A. japonicus* (PDB id: 3lfi.1) was used as a template.

### GH32 orthology relationships

Several methods were employed to establish the orthology relationships among the Ceratocystidaceae GH32 homologs. This was important as the characterization of homologous proteins/genes (i.e., those derived from a common ancestry) facilitates inferences regarding their evolution and function [35]. In this study, we used the definitions proposed by Koonin [36] for the terms “paralogy” and “orthology”. While orthologs (i.e., homologs that evolved from a common ancestor through speciation) are expected to encode proteins with equivalent functions, paralogs (i.e., homologs that are the product of an ancestral duplication) are thought to more readily acquire novel functional roles [36].

The orthology relationships among the Ceratocystidaceae GH32 homologs were predicted using phylogenetic criteria [34, 37]. For this purpose, a Maximum Likelihood (ML) phylogeny was constructed with the putative Ceratocystidaceae and Sordariomycetes GH32 members identified in this study, as well as the protein sequences of currently described members of family GH32 in the Carbohydrate-Active enZYmes (CAZY) database (http://afmb.cnrs-mrs.fr/CAZY/), which were obtained from the NCBI database. For this purpose, the sequences were aligned using MAFFT (Multiple sequence alignment based on fast Fourier transform) v.7.0 (http://mafft.cbrc.jp/alignment/software/) with the L-INS-i option [38]. Motifs that were not present in all of the sequences (e.g., eukaryotic signal motif for extracellular localization and transmembrane motifs for intracellular localisation) were excluded from the alignment. ML analysis was performed using PhyML v.3.0 [39] with the best-fit amino acid substitution model as indicated by ProtTest v.2.4 [40]. The GH32 family ML analysis incorporated the Le-Gascuel (LG) model [41], a proportion of invariable sites (I) and the observed amino acid frequencies (F). Branch support was estimated with PhyML using 1000 bootstrap replicates and the same best-fit models and parameters. Phylogenetic trees were viewed and edited using MEGA v.5 [42].

Gene order (i.e., synteny) and gene structure information was also used to investigate orthology relationships among the Ceratocystidaceae GH32 gene family members. According to Jun et al. [43] orthologous genes typically share homologous neighbouring genes, while non-orthologous genes are typically not flanked by homologous neighbours. Also, orthologous genes will more likely be structured similarly (i.e., share specific domains and introns) than non-orthologous genes [43]. For the gene order analyses, genes and proteins were predicted on all the scaffolds harbouring GH32 gene family members using AUGUSTUS [32]. The predicted genes were then annotated using Blast2GO [44] in the CLC Genomics Workbench 6.0.1 (CLC Bio, Aarhus, Denmark). The sequences of these predicted genes, on each side of the GH32 gene family members, were then used in local BLAST searches in BioEdit. Homology between neighbouring genes was defined as those with blastp and tblastn E-values < 10^−5^. Gene structure similarity was measured using the intron conservation ratio (ICR) between two intron-bearing genes [43]. The ICR between two homologous genes was calculated as the number of positionally homologous introns (i.e., introns that occur at the same position in different genes) divided by the total number of intron positions from the protein alignment [43]. Non-orthologous genes are expected to have ICR-values < 0.5 according to Jun et al. [43].

Finally, OrthoMCL v.2.0.9 [35] was used in an all-against-all BLAST search, followed by a Markov Cluster analysis to group putative orthologs and paralogs between the *Huntiella* and *Ceratocystis* species. For this analysis, we constructed a sequence database consisting of 43 052 predicted proteins, which consisted of all the AUGUSTUS-predicted proteins for each of the *Huntiella* and *Ceratocystis* species. OrthoMCL was run according to the recommended parameters, with an E-value threshold of 10^−5^ [35].

### Analysis of GH32 gene family evolution

To make inferences regarding GH32 gene family expansions and contractions across the fungi examined in this study, we employed CAFE v.3.1 (Computational Analysis of gene Family Evolution) [45]. For these analyses, the birth (λ) and death (μ) rates were estimated using the lambdamu tool with ‘-s’ option, while the number of gene gains and losses on each branch of the tree was estimated with the ‘-t’ option. The estimated birth and death rates (λ and μ) used in the subsequent analysis were 0.003 and 0.005, respectively. CAFE was run with default parameters of a *P*-value cut-off of 0.01 (option -p) and the number of random samples used the default value of 1000 (option -r). A time-calibrated Sordariomycetes tree (see below) was used in this analysis where transitions over individual branches were considered significant at *P*<0.005.

To generate the time-calibrated Sordariomycetes tree needed for the CAFE analysis, the Bayesian Evolutionary Analysis by Sampling Trees (BEAST) package v.2.2.1 [46] was used. For this purpose, we utilized five single copy genes routinely used for phylogenetic analyses [20, 47, 48]. The data (see Additional file 1: Table S1) for the analysis were extracted from the *Huntiella* and *Ceratocystis* genomes by performing local tblastn analysis (E-value < 10^−5^) in BioEdit using reference sequences from *A. clavatus*. These were elongation factor-1 alpha [EF-1a, GenBank:7000001156883129], elongation factor-3 alpha [EF3, GenBank:7000001156847434], mini-chromosome maintenance complex component 7 [MCM7, GenBank:7000001156824401], RNA polymerase II largest subunit [RPB1, GenBank:XP_001268791] and RNA polymerase II second largest subunit [RPB2, GenBank:XP_001272355)]. These respective gene sequences were also extracted from the representative Sordariomycetes included in the JGI database. The relevant sequences for outgroup taxa in the Dothideomycetes (*Alternaria brassicicola, Stagnospora nodrum* and *Mycosphaerella fijiensis*) were also obtained using the JGI portal.

The five protein sequences were aligned with MAFFT as described above and the alignment served as input for a Bayesian tree search with BEAST. A ProtTest analysis suggested the Whelan and Goldman (WAG; [49]) model as the best-fitting evolutionary model for this data. To generate a time-calibrated tree, the analysis was run using the Markov chain Monte Carlo (MCMC) method and four calibration points, which included the Dothideomycetes crown group (mean 350 Million years ago [Mya] with a 95 % credibility interval [CI] of 273–459) [50], the last common ancestor (LCA) of the Hypocreales (181 Mya with a 95 % CI of 150–213) [51], the Clavicipitaceae crown group (117 Mya with a 95 % CI of 95–144) [51], as well as the Nectriaceae crown group (125 Mya with a 95 % CI of 98–155) [51, 52]. The program BEAUTi v.2.0 was used to prepare an xml file to create a starting tree for the BEAST analyses. Priors included the strict molecular clock model with a Yule process for the model of speciation [53]. The standard deviation of all distributions was set to 1.0. Two analyses were run with 10,000,000 generations, sampling data every 1000th generation. The first 15 % of the trees were removed (burn-in) and a consensus of the remaining trees was obtained using LogCombiner and TreeAnnotator [46] and viewed using FigTree v.1.3.1 (http://tree.bio.ed.ac.uk/software/figtree). Tracer v.1.5 (http://beast.bio.ed.ac.uk/Tracer) was used to inspect the chains for convergence, and to ensure that ESS (Effective Sample Size) values exceeded 200 [46].

### *Fot5* analysis

The genomic distribution of *pogo*-like elements, which are homologous to *F. oxysporum* transposase 5 (*Fot5*; [54]) in the Ceratocystidaceae, were investigated, as this element was located near the GH32 family genes in the genomes of the *Ceratocystis* species examined. For this purpose, the *F. oxysporum Fot5* protein sequence [GenBank: AJ608703] was used in local BLAST searches (tblastn E-value < 10–5) with BioEdit to identify homologs in the *Huntiella* and *Ceratocystis* genomes. The conserved DDD catalytic domain of *Fot5* (i.e., triad of acidic amino acids [Asp-Asp-Asp or Asp-Asp-Glx] that forms the catalytic pocket for the cleavage of DNA strands) [55] of the homologs identified here, and the previously characterised *pogo*-like transposons [56] were aligned with MAFFT as described above. This alignment was subjected to ML tree reconstruction using PhyML with the best-fit model parameters (WAG plus gamma to account for among site rate variation) as indicated by ProtTest. Branch support was estimated with PhyML using 1000 bootstrap replicates and the same model parameters.

Whether the *Fot5* homologs identified in *Ceratocystis* have been subjected to repeat-induced point mutation (RIP) was also considered. In filamentous fungi, RIP is a defense mechanism against mobile genetic elements [56] and involves the transition from C:G to T:A nucleotides in pairs of duplicated sequences during meiosis [57]. Therefore, the TpA/ApT ratio across the various *Ceratocystis Fot5* sequences was measured. This simple index reflects the frequency of TpA RIP products, and was used as an indication of the RIP response [58]. We also calculated the (CpA + TpG)/(ApC + GpT) index, which considers both the products (TpA) and the targets (CpA and TpG) of RIP [58]. RIPCAL (http://www.sourceforge.net/projects/ripcal) was used to calculate these indices in the aligned *Fot5* nucleotide sequences of *Ceratocystis*.

## Results

### Genome sequences

Illumina sequencing of the *H. savannae* isolate produced a total of 2 884 747 186 bases of trimmed reads with an average length of 85.68 bases (Table 2). The draft genome of this isolate contained 28.54 megabases (Mb) and was made up of 361 scaffolds larger than 500 bases, of which the largest was 1 009 760 bases in length (Table 2). The assembly had an N50 scaffold size of 229 095 bases with a GC content of approximately 47.39 %. The *H. savannae* draft genome assembly was predicted to encode 7 687 putative ORFs with CEGMA completeness scores of 96.37 % (partial), which is comparable to the draft genomes of *H. omanensis* (31.5 Mb in size and encodes 8 395 ORFs, [27]), *H. moniliformis* (25 Mb in size and encodes 7000 ORFs, [26]), *C. fimbriata* (29.4 Mb in size and encodes 7 266 ORFs), [21], *C. albifundus* (27.2 Mb in size and encodes 6 967 ORFs, [27]) and *C. manginecans* (31.7 Mb in size and encodes 7 494 ORFs, [26]).

**Table 2:** Statistics of the *Huntiella savannae* genome assembly and gene annotations

Summary data	*Huntiella savannae*
Total reads before trim (bp)	33 168 540
Total reads after trim (bp)	33 055 449
Average length of reads before trim (bp)	101
Average length of reads after trim (bp)	85.68
Number of scaffolds	361
Total sequence length (Mb)	28.54
Largest scaffold (bp)	1 009 760
N50 Scaffold size (bp)	229 095
GC %	47.39
Predicted gene models	7 687
CEGMA^a^	96.37

^a^ Genome completeness was evaluated using the Core Eukaryotic Genes Mapping Approach (CEGMA) pipeline [38]

With the exception of *C. fimbriata*, the Ceratocystidaceae GH32 gene family members were all located on single contigs (*C. manginecans*: scaffold JJRZ01000038; *C. albifundus*: scaffold JSSU01001085; *H. omanensis*: scaffold JSUI01006495; *H. savannae*: scaffold NODE_2; *H. moniliformis*: scaffold JMSH01000004) (see Table 3 for gene locations and sizes). The GH32 genes of *C. fimbriata* were located on two scaffolds (*C. fimbriata*: scaffold APWK02000925 and scaffold APWK02000924). To join the two *C. fimbriata* scaffolds, as well as extend the scaffolds harbouring these genes in *C. fimbriata* and *C. albifundus* we employed the option ‘Map Reads to Reference’ in the CLC Genomics Workbench (mismatch cost = 2, insertion and deletion cost = 3, length fraction = 1.0, similarity fraction = 0.9) using scaffold JJRZ01000038 of *C. manginecans* as a reference. Scaffolds that harboured the GH32 genes in *H. savannae* and *H. omanensis* were similarly extended using scaffold JMSH01000004 of *H. moniliformis* as reference. The *C. manginecans* and *H. moniliformis* scaffolds were selected as references because these assemblies were most complete (Table 1) and also had the longest scaffolds that contained the GH32 genes.

**Table 3:** GH32 family members identified in *Huntiella* and *Ceratocystis*

Gene Name	Taxon^a^	Genomic Location^b^	Length (bp)	Length (aa)
CmINV_V	*Ceratocystis manginecans*	JJRZ01000038:33284-34837	1945	627
CmINV_CW	*Ceratocystis manginecans*	JJRZ01000038:36427-37962	1952	625
CfINV_V	*Ceratocystis fimbriata*	APWK02000925:4324-5817	1945	627
CfINV_CW	*Ceratocystis fimbriata*	APWK02000924:3-1490	1938	625
CaINV_V	*Ceratocystis albifundus*	JSSU01001085:20903-22444	1829	627
CaINV_CW	*Ceratocystis albifundus*	JSSU01001085:17498-19033	1952	625
HmINV_CW	*Huntiella moniliformis*	JMSH01000004:78747-80591	1848	615
HaINV_CW	*Huntiella savannae*	NODE_2:199823- 201667	1848	615
HoINV_CW	*Huntiella omanensis*	JSUI01006495:45419-47263	1848	615

^a^ The GenBank accesssion numbers for the *C. manginecans*, *C. fimbriata* and *C. albifundus* genomes are JJRZ01000000, APWK00000000 and JSSU00000000. Those for the *H. moniliformis*, *H. savannae* and *H. omanensis* genomes are JMSH00000000, LCZG00000000 and SUI00000000

^b^ The name of the contig is followed by the nucleotide position of the gene within the contig

### GH32 gene identification and characterisation

**Fig. 1:**
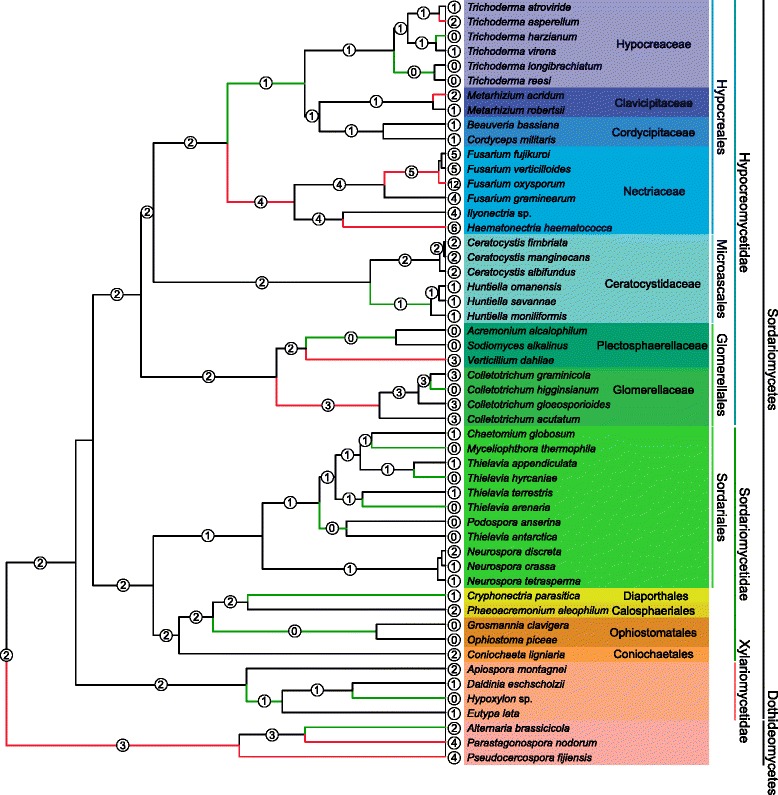
Glycoside hydrolase 32 (GH32) gene family expansions and contractions mapped onto the Sordariomycetes chronogram. Significant (*P*< 0.05) expansions (indicated with red lines) and contractions (indicated with green lines) were inferred using CAFE v3.1 (Computational Analysis of gene Family Evolution) [45]. The probable ancestral gene family size for each node is indicated within white circles, while the family sizes in extant species are indicated at the tips of terminal branches. Colour-coding designates the Sordariomycetes taxa to either order or family level. The chronogram was inferred in this study (see Additional file 2: Figure S1). The sequences from Dothideomycetes were used for outgroup purposes

All of the *Ceratocystis* and *Huntiella* genomes investigated in this study contained at least one putative member of the GH32 family. For the Ceratocystidaceae, the *Huntiella* species each contained a single copy of the gene (designated as *HaINV*-CW, *HsINV*-CW and *HmINV*-CW [previously named as *CmINV;* [28]), while the *Ceratocystis* species each contained two copies (designated as *CaINV*-CW, *CaINV*-V, *CfINV*-CW, *CfINV*-V, *CmINV*-CW and *CmINV*-V) (Fig. 1; Table 3). The distribution of the GH32 family genes among other Sordariomycetes varied greatly and some taxa lacked a GH32 gene altogether (Fig. 1). For example, 0–4 genes were identified in the Xylariales, 0–12 in the Hypocreales, and 0–3 in the Sordariales and Glomerellales. As expected [2], the plant pathogens generally contained more GH32 genes (e.g., 12 in *F. oxysporum*; and 6 in each of *A. terreus*, *Talaromyces stipitatus* and *Nectria haematococca*). These genes also appeared to be absent from insect pathogens and plant pathogens that evolved from insect pathogens (e.g., Cordycipitaceae and Clavicipitaceae) [47].

**Fig. 2:**
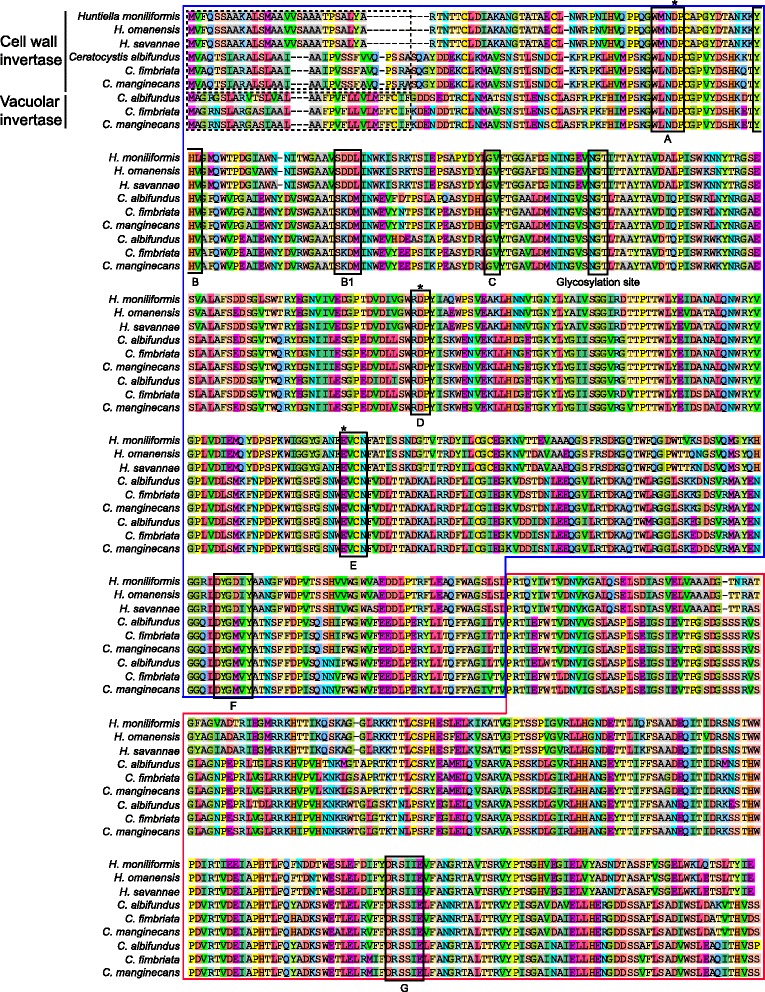
Alignment of the conserved motifs of the glycoside hydrolase 32 (GH32) enzymes. These include conserved regions (labelled A-G) and various amino acids (shown with black stars). The N-terminal β-propeller module (indicated in the blue block) and the C-terminal β-sandwich module (indicated in the red block) are also highlighted. The translated sequences of one group of the *Ceratocystis* GH32 gene possess a trans-membrane domain (shown with dotted lines) characteristic of vacuolar invertases [5], while the translated sequences of the other *Ceratocystis* GH32 gene and the *Huntiella* GH32 gene possess an eukaryotic secretion signal (shown with dotted lines) needed for secretion [60]

**Fig. 3:**
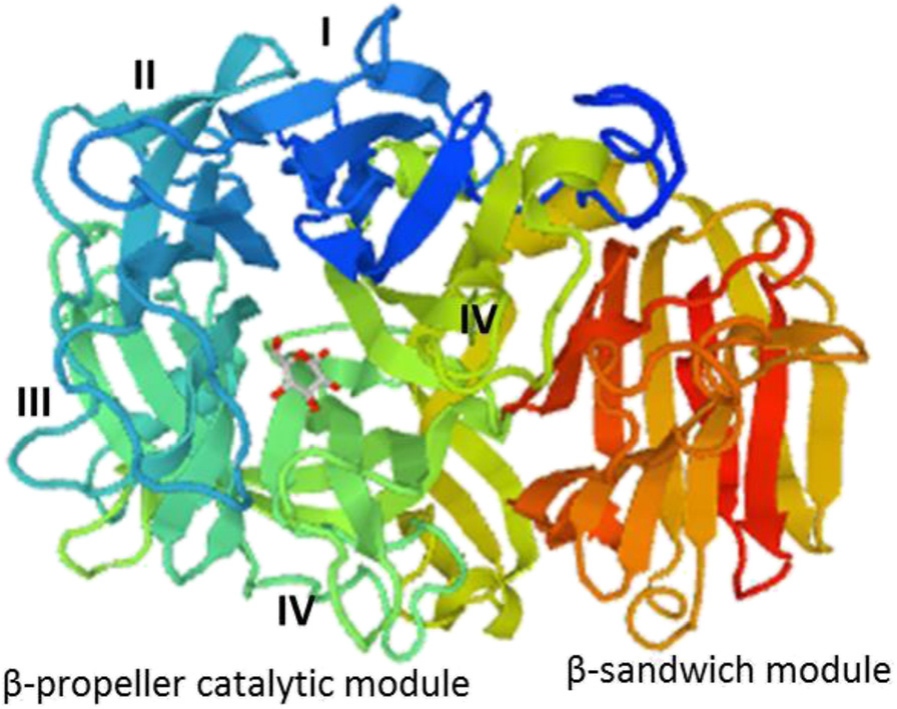
3D structure of the *C. manginecans* invertase (CmINV-CW). Roman numerals (I–V) show the five blades of the β-propeller module, while the C-terminal β-sandwich module is indicated in dark red. These structures were inferred with the Swiss-Model Web server (http://www.expasy.org/swissmod/SWISS-MODEL.html) by making use of a fructosyltransferase from *Aspergillus japonicus* (PDB id: 3lfi.1) as template

Among the examined Ceratocystidaceae GH32 family members, the InterProScan and SignalP analyses identified a conserved N-terminal (IPR013148) (Fig. 2). These analyses also identified a less conserved C-terminal (IPR013189) that is likely essential for overall protein stability ([59]; Fig. 2). These sequences, including those identified for the Ceratocystidaceae, also contain the eight well-conserved domains (A-G) and three highly conserved acidic residues characteristic of GH32 gene family members ([60]; Fig. 2). The latter include an aspartic acid located in the WMNDPNG motif (also known as β-fructosidase motif or sucrose-binding box) of domain A that acts as a nucleophile, and an aspartic acid located in the RDP motif of domain D that acts as a transition-state stabiliser, as well as a glutamic acid located in the EC motif of domain E that acts as the acid/base catalyst [3]. Compared to the NG present in the WMNDPNG motif of other fungi [57], the *Huntiella* GH32 genes contain a CA, while those of the *Ceratocystis* genes contain a CG. NetNGlyc analysis also revealed that all of the Ceratocystidaceae GH32 genes contained a potential N-glycosylation site. This indicates that the genes identified in these fungi encode a conserved asparagine residue that is predicted to attach to a glycan chain to facilitate various co- and post-translational modifications and enhance the stability, transport and secretion of proteins [61, 62]. The inferred 3D structures of the proteins encoded by Ceratocystidaceae GH32 genes further confirmed the presence of the five-bladed β-propeller catalytic module at the N-terminal, as well as the presence of two six-stranded β-sheets composed of antiparallel β-strands forming a sandwich-like fold at the C-terminal domain (Fig. 3).

The SignalP analyses showed that parts of the inferred amino acid sequences of the *Huntiella* genes (i.e., the first 28 residues encoded by *HaINV*-CW, *HsINV*-CW and *HmINV*-CW), as well as one of the *Ceratocystis* homologs (i.e., the first 31 residues encoded by *CaINV*-CW, *CfINV*-CW and *CmINV*-CW) are comprised of a eukaryotic secretion signal. This suggests an extracellular localisation for the proteins, which is typical of cell wall invertases [16]. These analyses also predicted possible signal peptide cleavage sites between amino acids 25 and 26 for the *Huntiella* homologs and between residues 19 and 20 for the one *Ceratocystis* homolog (Fig. 2). However, the second homolog of the gene in *Ceratocystis* species lacked the N-terminal signal sequence. Instead, parts of the translated sequences of this gene (i.e., the first 32 residues encoded by *CaINV*-V, *CfINV*-V and *CmINV*-V) comprised a transmembrane region, which is characteristic of vacuolar invertases [5] suggesting an intracellular localisation for the protein. Our analysis also suggested that this homolog adopts the NinCout configuration that consists of a short N-terminal segment in the cytosol and a long C-terminal region in the vacuole, which is typical of MEnM of type II single-pass membrane proteins [5]. We therefore classified the Ceratocystidaceae GH32 gene family homologs as either cell wall invertases (with a CW suffix to gene and protein names; for the *Huntiella* homologs and one group of homologs in *Ceratocystis*), or as vacuolar invertases (with a V suffix to gene and protein names; for the second homolog in *Ceratocystis*).

The SignalP analyses of GH32 gene family members in the other Sordariomycetes showed that genes belonging to the groups designated by Parrent et al. [2] as extracellular invertases contained the eukaryotic secretion signal motif. In contrast, this motif was absent from genes that belonged to the groups they designated as intracellular invertases. Indeed, previous molecular and biochemical studies have shown that the eukaryotic secretion signal motif is present in genes encoding extracellular invertases and absent from genes encoding intracellular invertases [63, 64]. Except for the three *Ceratocystis* genes (i.e., *CaINV*-V, *CfINV*-V and *CmINV*-V), none of the other Sordariomycetes GH32 genes contained the transmembrane motif, which is characteristic of vacuolar invertase genes.

### GH32 orthology relationships

**Fig. 4:**
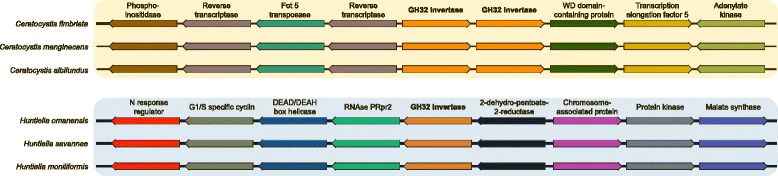
The predicted genes flanking the Glycoside hydrolase 32 (GH32) gene family members in *Huntiella* and *Ceratocystis*. Genes present on the scaffolds harbouring the putative invertases were predicted using AUGUSTUS [32] and annotated using Blast2GO [44]. Note that the genes are not drawn to scale. The *Huntiella* GH32 family gene is flanked by putative G1/S-specific cyclin Pcl5 (*Colletotrichum orbiculare*, ENH86823), RNAse P Rpr2/Rpp21 subunit domain-containing protein (*Gaeumannomyces gramini*, EPQ63823), Malate synthase-like protein (*Acremonium chrysogenum*, XP003651419), serine/threonine-protein kinase (*Metarhizium acridum*, EFY93082.1), nitrogen response regulator (*Colletotrichum gloeosporioides*, ELA29612.1), DEAD/DEAH box helicase (*Colletotrichum sublineola*, KDN64774), 2-dehydropantoate 2-reductase (*Colletotrichum gloeosporioides*, EQB48758), and structural maintenance of chromosomes 5 (*Villosiclava virens*, KDB17190) genes. The two *Ceratocystis* GH32 family genes were flanked by putative Phosphatidylinositol-specific phospholipase (*Metarhizium anisopliae*, KFG82763), putative WD domain-containing protein (*Togninia minima*, EOO00810.1), reverse transcriptases (*Sclerotinia sclerotiorum*, XP_001588999 and *Blumeria graminis*, CCU77161), transcription elongation factor 5 (*Scedosporium apiospermum*, KEZ42236), adenylate kinase (*Magnaporthe oryzae*, XP003716198), and *Fot5* transposase (*Colletotrichum gloeosporioides*, ELA33194.1) genes

Gene order analysis of the *Ceratocystis* scaffolds harbouring GH32 family members revealed that the cell wall and vacuolar invertase genes are located adjacent to each other in all three of the species studied. However, the *Huntiella* cell wall invertase gene is located at a different genomic region when compared to that of *Ceratocystis* (Fig. 4). This was confirmed using the gene order analysis, where homologous flanking genes (tblastn, E-values < 10^−5^) were only obtained for the within-genus comparisons. Genes encoded on the examined scaffolds, other than the GH32 family genes, had homologs elsewhere in the genomes of the two fungi (e.g. the *Ceratocystis* scaffolds harboured various putative reverse transcriptase genes, which were also present on scaffolds other than the one harbouring the GH32 gene in the *Huntiella* genomes). An exception was for the *Fot5* transposase genes that were only present in the genomes of the *Ceratocystis* species (see below). According to Jun et al. [43], such an observed lack of synteny points towards a non-orthologous relationship between the GH32 genes of *Ceratocystis* and *Huntiella*.

Analysis of gene and protein structures of the Ceratocystidaceae and Sordariomycetes GH32 family members revealed that coding sequences were interrupted by introns that vary greatly in number and distribution across all of the taxa examined in this study (Fig. 2). For example, the *Huntiella* genes (consisting of 1 848 bases and encoding 615 aa) did not harbour any introns, while both the *Ceratocystis* genes (consisting of 1 945–1 952 bases and encoding 625–627 aa) contained a single intron at the same position (Table 3). This corresponded to an ICR of 1 for the *Ceratocystis* GH32 family members, and an ICR value of 0 for the Ceratocystidaceae GH32 family members. According to Jun et al. [43], the latter ICR value indicates non-orthology between the GH32 genes of *Ceratocystis* and *Huntiella*.

**Fig. 5:**
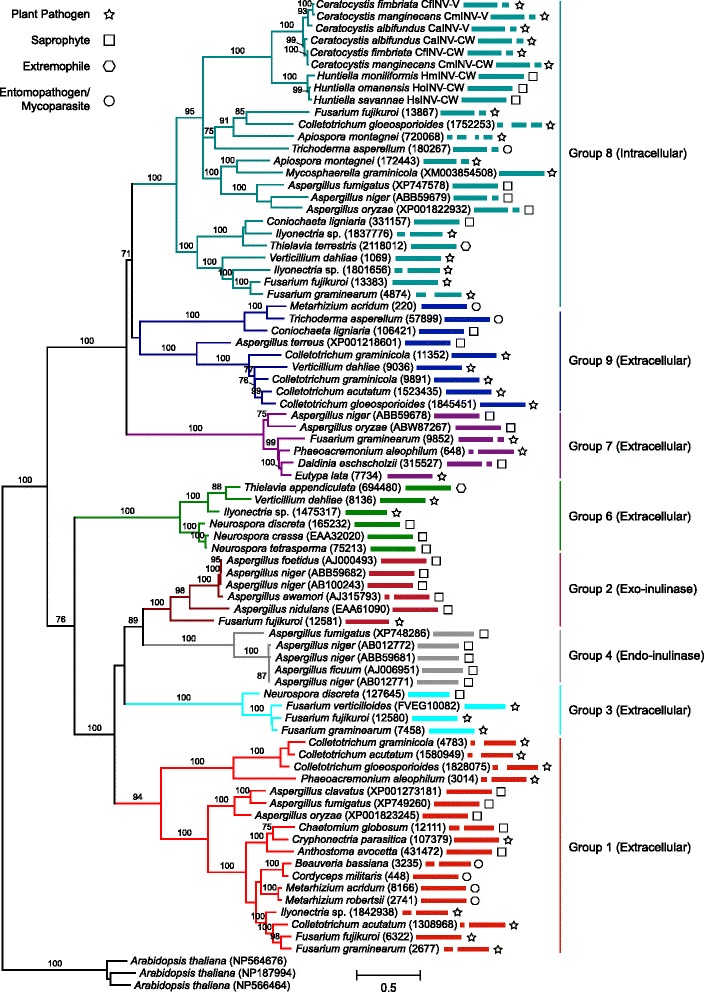
Maximum likelihood phylogeny of the Sordariomycetes Glycoside Hydrolase 32 (GH32) gene family. Representative sequences of the 8 groups that span the fungal GH32 gene phylogeny [2] were included in this study. GenBank accession numbers or sequence identifiers from genome projects for each of these sequences are provided in parentheses. Percentage bootstrap support (based on a 1000 repeats) is indicated at the internodes. The exon-intron structure of the genes is diagrammatically indicated next to each taxon where gaps within solid lines indicate intron positions. Colour-coding designates the groups previously identified [2]. The sequences from *Arabidobsis thaliana* were used for outgroup purposes

**Fig. 6:**
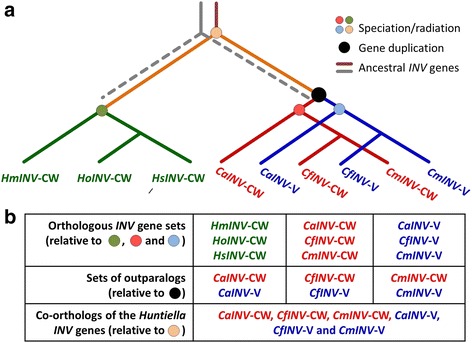
The inferred evolutionary history of the Ceratocystidaceae Glycoside hydrolase 32 (GH32) gene family and the orthology relationships among these genes. **a** The evolutionary tree shows nine homologous genes from six species (A). The *Huntiella* cell wall invertase genes are depicted as *HsINV*-CW, *HmINV*-CW and *HoINV*-CW, while the *Ceratocystis* vacuolar invertase genes are depicted as *CaINV*-V, *CmINV*-V and *CfINV*-V and those encoding the *Ceratocystis* cell wall invertases as *CaINV*-CW, *CmINV*-CW and *CfINV*-CW. As indicated by CAFE, the genome of the Ceratocystidaceae ancestor likely encoded two invertase (*INV*) genes, one of which (depicted by the grey line) was subsequently lost from both the *Ceratocystis* and *Huntiella* lineages (depicted by grey broken line) before the radiation of species. However, the remaining invertase gene (depicted in orange) was duplicated in the *Ceratocystis* ancestor resulting in the two invertase genes encoded by the genomes of the extant species. This duplication was also reconstructed using NOTUNG 2.6 which detects duplications based on gene tree to species tree reconciliation [89] (results not shown). All of the invertase genes in the extant *Ceratocystis* and *Huntiella* species thus evolved from the same ancestral gene in the Ceratocystidaceae ancestor (depicted by the orange line). The respective *Ceratocystis* genes each evolved through vertical decent after their emergence (i.e., gene duplication) in the last common ancestor. **b** Following the standard nomenclature for duplicated genes (reviewed by Koonin [36]), the *Huntiella* cell wall invertase genes share an orthologous relationship (i.e., orthologs are related via speciation and are derived via vertical decent from the common ancestor). The same is also true for the respective cell wall and vacuolar invertase genes of *Ceratocystis*, where each represent a set of orthologs. Because the duplication that gave rise to the *Certocystis* genes occurred before radiation of this genus, the *Ceratocystis* cell wall and vacuolar invertase genes represent outparalogs (i.e., homologs that derive from a gene duplication event that precedes lineage radiation/speciation) [36]. However, all of the *Ceratocysistis* invertase genes represent co-orthologs of the gene in *Huntiella*. This is because the lineage-specific duplication in *Ceratocystis* gave rise to a set of genes that are collectively orthologous to those of *Huntiella* [36]

The ML phylogeny revealed that the *Huntiella* and *Ceratocystis* GH32 genes grouped with known members of this protein family (Fig. 5). The *Ceratocystis* vacuolar invertases formed part of a well-supported clade previously designated as Group 8 [2], which include invertases with intracellular localisation and that lack signal peptide cleavage sites (Fig. 5). Despite the presence of signal peptides for extracellular localisation, however, the *Huntiella* and *Ceratocystis* cell wall invertases also formed part of Group 8. Within this clade, the Ceratocystidaceae genes grouped according to their evolutionary relationships (i.e., the two genes in *Ceratocystis* were more closely related to each other than to the gene in *Huntiella*). Within *Ceratocystis*, the cell wall invertases formed a sister group to the vacuolar invertases; and within each of these sister groups, the relationships among the genes matched the known relationships among species, with the sequences of *C. fimbriata* and *C. manginecans* grouping together and *C. albifundus* at their base. The same was also true for the *Huntiella* cell wall invertase genes. Therefore, *CaINV*-V, *CfINV*-V and *CmINV*-V are orthologs, *CaINV*-CW, *CfINV*-CW and *CmINV*-CW are orthologs and *HaINV*-CW, *HsINV*-CW and *HmINV*-CW are orthologs (Fig. 6) [34]. The *Ceratocystis* GH32 genes represent co-orthologs of the *Huntiella* genes (i.e., the two *Ceratocystis* GH32 genes are collectively orthologous to the *Huntiella* GH32 gene due to a lineage-specific duplication in the former, Fig. 6) [34]. Because the duplication that gave rise to the two *Ceratocystis* GH32 genes occurred in the ancestor of this genus, the cell wall and vacuolar invertase genes of these species represent outparalogs (i.e., paralogous genes derived from a gene duplication event that precedes lineage radiation [34], Fig. 6). These orthology relationships were consistent with the results of the OrthoMCL analysis. Therefore, the non-orthology of the GH32 genes in *Ceratocystis* and *Huntiella,* suggested by the results of the synteny and ICR analyses, likely reflects the involvement of retrotransposition in the evolution of these genes (see below).

### GH32 gene family evolution

BEAST and CAFE analyses were used to identify and estimate the relative ages of the losses/gains of the GH32 family genes in several orders and families in the Sordariomycetes, including Ceratocystidaceae (Fig. 1, Additional file 2: Figure S1). The ESS-values for the BEAST analysis parameters were higher than 200, which is the recommended threshold for ensuring appropriate estimation of the posterior distribution of each parameter [46]. As expected from the analysis, the root node that represents the divergence of the Sordariomycetes and Dothideomycetes was around 362 Mya (with CI of 346–377 Mya) [51–65]. Based on these data, the estimated divergence time for the LCA of *Huntiella* and *Ceratocystis* was *ca*. 62 Mya (with CI of 50–70 Mya).

The CAFE analysis identified several gene loss and gain events in the GH32 gene family (Fig. 1). Many of these were inferred to be lineage-specific, which included significant expansions (e.g., *F. oxysporum* with 12 gene copies and *N. haematococca* with 6 gene copies) and contractions (e.g., *Hypoxylon* sp., *Thielavavia arenaria*, *Myceliophthora thermophila*, and *Colletotrichum higginsianum* all lacking GH32 family members) at the tips of branches. At deeper phylogenetic levels, significant expansions were predicted for branches leading to the Nectriaceae and the outgroup taxa in the Dothideomycetes, while significant contractions were predicted for branches leading to the Sordariales, Ophiostomatales, Xylariales, as well as the branch leading to Hypocreaceae, Clavicitpitaceae and Cordycipitaceae. Among the Ceratocystidaceae, a GH32 family contraction was predicted for the *Huntiella* species (*ca*. 62 Mya). Other GH32 family contractions and expansions in the Sordariomycetes predicted for the first time in the current study include an expansion on the branch leading to the Glomerellaceae and an expansion on the branch leading to the Nectriaceae, as well as a contraction on the branch leading to the Hypocreaceae-Clavicipitaceae-Cordycipitaceae clade.

### *Fot5* analysis

**Fig. 7:**
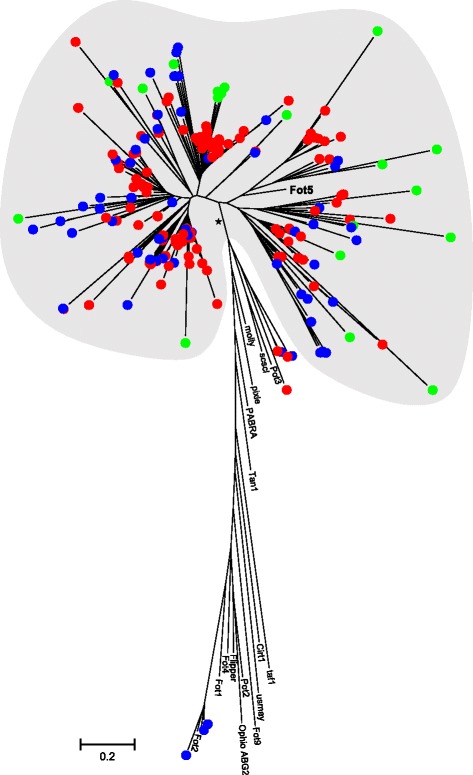
Maximum likelihood phylogeny of the *Fot5* DDD catalytic domain. This analysis was done using the WAG substitution model [49] and gamma correction to account for among site rate variation. The *Ceratocystis Fot5* sequences are included in the grey area and indicated according to species (green dots = *C. albifundus*, blue dots = *C. fimbriata*, red dots = *C. manginecans*). The branch labelled with an asterisk received 81 % bootstrap support based on the analysis of 1000 pseudoreplicates (see Additional file 4: Figure S2 for full information regarding bootstrap support for the tree, as well as the sequence identifiers of putative *Ceratocystis Fot5* homologs and Additional file 3: Table S2 for their genomic coordinates). GenBank accession numbers or for previously identified *Fot5* homologs are: Fot2 [Genbank:JN624854, *F. oxysporum*), *Fot5* [Genbank:CAE55867, *F. oxysporum*], Fot1 [Genbank:X64799, *F. oxysporum*], Fot4 [Genbank:AF076632, *F. oxysporum*], Fot9 [JGI:2517, *F. graminearum*], Fotyl [Genbank:CAG33729.1 *Yarrowia lipolytica*], Molly [Genbank:CAD32687, *Parastagonospora nodorum*], Ophio [Genbank:ABG26269, *Ophiostoma novo-ulmi*], PABRA [Genbank:ACY56713, *Paracoccidioides brasiliensis*], Pixie [Genbank:CAD32689, *Parastagonospora nodorum*], Pot2 [Genbank:CAA83918, *Magnaporthe grisea*], Pot3 [Genbank:AAC49418, *M. grisea*], SCSCL [Genbank:XP001592252, *Sclerotinia sclerotiorum*], Taf1 [Genbank:AAX83011, *Aspergillus fumigatus*], Tan1 [Genbank:U58946, *Aspergillus awamori*] USMA [Genbank:UM03882, *Ustilago maydis*), Flipper [Genbank:AAB63315, *Botryotinia fuckeliana*] and Cirt1 [Genbank:XP710204, *Candida albicans*]

Local BLAST searches with the *F. oxysporum Fot5* sequence revealed that this gene family is apparently absent from the *Huntiella* genomes, while the *Ceratocystis* genomes harbour numerous *Fot5* homologs (Additional file 3: Table S2). Phylogenetic analysis of the 202 sequences (i.e., 60 from *C. fimbriata*, 19 from *C. albifundus* and 106 from *C. manginecans*, as well as 17 previously characterised pogo-like transposon sequences) spanning more than 75 % of the DDD catalytic domain of *Fot5*, confirmed that most of these sequences indeed represent putative *Fot5* homologs (Fig. 7 Additional file 4: Figure S2). The identified *Ceratocystis Fot5* sequences formed a monophyletic group with the known *Fot5* sequence from *F. oxysporum* with high bootstrap support (81 %) (Fig. 7 Additional file 4: Figure S2). However, some sequences of *Ceratocystis* also clustered together with the other *Fot* family members: three copies of *C. fimbriata* clustered with *Fot2*, two copies of *C. fimbriata* and three copies of *C. manginecans* clustered with *Pot3* and SCSCL. Several groups of identical and closely related *Fot5* homologs were detected, where homologs belonging to the same species and homologs belonging to different species often grouped together.

The putative *Fot5* homologs identified in the *Ceratocystis* genomes displayed the hallmarks of RIP. Overall, the *Fot5* sequences had TpA/ApT index values above 1 (1.5 for *C. albifundus*, 1.3 for *C. fimbriata* and 1.5 for *C. manginecans*), possibly due to the introduction of C:G to T:A mutations [58]. The *Fot5* sequences also had lower (CpA + TpG)/(ApC + GpT) index values (1.2 for *C. albifundus*, 1.1 for *C. fimbriata* and 1.3 for *C. manginecans*), indicating a possible RIP response [58]. Analysis of individual sequences revealed a mixture of RIPped and non-RIPped copies, with 56 % of the *C. albifundus Fot5* homologs, 35 % of the *C. fimbriata Fot5* homologs and 32 % of the *C. manginecans Fot5* homologs having TpA/ApT ratios of >1 and A + T richness > 55 % [56]. According to Dufresne et al. [56] this is indicative of a mild RIP response, allowing the presence of potentially active *Fot5* copies.

## Discussion

All of the identified Ceratocystidaceae invertase genes and inferred proteins carry hallmarks of the GH32 gene family and were considered homologs. They all have an N-terminal catalytic domain and a C-terminal β-sandwich domain needed for structural stability [9]. They also contained three conserved residues (i.e., two aspartates and one glutamate) referred to as ‘the catalytic triad’ (see Fig. 2), which are indispensable for binding and catalysis [3, 5]. For example, it was suggested that the aspartate present in the RDP-motif provides hydrogen bonds to bind the C3 and C4 hydroxyls of fructose [3]. Although the WMNDPNG-motif present in the Ceratocystidaceae invertases is not fully conserved, they do contain the two critical amino acids (W and N) needed for transfructosylation [66]. Typical of vacuolar and cell wall invertases, all of the Ceratocystidaceae sequences also contained an N-glycosylation site where a glycan chain can potentially attach to an asparagine residue of the acceptor proteins [67]. Given these commonalities with other GH32 enzymes, it is likely that the invertases encoded by the Ceratocystidaceae represent active enzymes with sucrolytic activities. Thus far, heterologous expression of the *HmINV*-CW gene of *H. moniliformis* in *S. cerevisiae* yielded an active invertase that allowed the mutant yeast to utilize sucrose as sole carbohydrate source [28]. However, further studies are required to determine if both the vacuolar and cell wall invertase genes identified in this study are functional in all of the Ceratocystidaceae that harbour them.

Most functional studies of fungal cell wall invertases have focused on industrial applications [14, 68], and very little is known regarding the biological functions of these enzymes. It is possible that the cell wall and vacuolar invertases of *Huntiella* and *Ceratocystis* may enable colonization of plant tissue by facilitating uptake and transport of plant-derived sucrose [62]. Previous studies have shown that during plant-fungus interactions, both partners contribute to the overall invertase activity [69]. Plants use invertases for sugar signalling linked to stress and defence responses in addition to nutrition, whereas, fungal invertases convert extracellular and intracellular sucrose to fructose and glucose, and ensure the availability of nutrients during infection [70–72]. These enzymes may also be involved in glucose signalling that may influence fungal virulence [73]. In these fungi, vacuolar invertases may streamline sucrose utilization, especially if the sucrose-cleaving activity becomes rate-limiting for provision of sugars to the fungus during infection [71]. The functional expression of GH32 enzymes in interactions between Ceratocystidaceae and their plant hosts and substrates, should be investigated to provide insights into the potential role this gene family plays in the infection biology and pathogenesis of this group of fungi.

To the best of our knowledge, these are the first vacuolar invertases identified in fungi. It is conceivable that gene duplication followed by functional divergence of the outparalogs gave rise to the two types of invertases in the Ceratocystidaceae (see Fig. 6). In fact, gene duplication followed by functional divergence have been shown to be important drivers of the evolution of GH families [74]. For example, small changes in the primary structure of GHs can result in changes to their substrate specificities [75], while changes at their N-terminals might influence cellular localisation [8]. Such changes at the N-terminal could have allowed for the evolution of the Ceratocystidaceae cell wall invertases from ancestral Group 8 intracellular invertases. Consistent with this view, the cell wall invertases of *Ceratocystis* and *Huntiella* both contain eukaryotic signal sequences for directing proteins into the endoplasmic reticulum for secretion [5, 76]. It is also consistent with previous predictions that *HmINV-CW* in *H. moniliformis* represents an extracellular invertase [28]. In turn, the vacuolar invertase of *Ceratocystis* could have evolved from a cell wall invertase as has previously been suggested for plant invertases [5]. Such a process would be facilitated by the loss of the eukaryotic secretion signal sequence and acquisition of signature motifs, which in plants allow for localisation to the lytic vacuole [5]. Indeed, structural analysis suggested that the putative vacuolar invertases of *Ceratocystis* adopt the characteristic NinCout configuration of type II single-pass membrane proteins that are targeted to vacuoles [5]. These data, together with the results of our phylogenetic analysis, strongly suggest that the evolution of the two invertase outparalogs in *Ceratocystis* involved divergence from a common ancestor by the loss and gain of motifs at their N-terminals to ultimately yield a cell wall and a vacuolar invertase.

The evolutionary history of the GH32 gene family in the Ceratocystidaceae was studied in CAFE by reconstruction of ancestral states across the Sordariomycetes. This approach involves an evaluation of the probabilities of changes in family size (i.e., gene copy number expansions and contractions) from “parent to child nodes” in a time-calibrated phylogeny [77]. The CAFE analysis showed that the LCAs of most of the Sordariomycetes orders, as well as the subclass Hypocreomycetidae, likely encoded two GH32 genes (i.e., a gene family size of two represents the ancestral or plesiomorphic state for these groups) (see Fig. 1). This was also true for the Ceratocystidaceae, where the only significant transition (a contraction) in GH32 gene family size occurred approximately 62.0 Mya in the LCA of *Huntiella*. However, based on the GH32 gene phylogeny, the Ceratocystidaceae invertases represent a nested and monophyletic cluster within GH32 Group 8, suggesting that all of the invertases in this fungal family evolved from a single ancestral gene (i.e., the *Ceratocystis* genes are collectively co-orthologous to the *Huntiella* GH32 gene). The most parsimonious explanation for these findings is therefore that the evolution of the Ceratocystidaceae GH32 gene family involved the loss of one of the two ancestral genes predicted by CAFE (i.e., one of the two GH32 genes predicted to have been encoded by the LCA of the Ceratocystidaceae was lost from both the *Ceratocystis* and *Huntiella* lineages) (Fig. 6). On the *Huntiella* branch, the remaining gene gave rise to the extant GH32 gene in this genus. In the LCA of *Ceratocystis*, a lineage-specific duplication of the remaining ancestral gene gave rise to the two GH32 genes of the extant species (Fig. 6). This duplication in the LCA of *Ceratocystis* also established a membership of two for its GH32 gene family. This superficially resembles the inferred ancestral state for the overall family, but the data clearly showed that the extant condition of having two GH32 genes emerged in the LCA of *Ceratocystis*, thus indicating that it represents the synapomorphic state for the genus.

The GH32 gene duplication in the *Ceratocystis* LCA likely allowed for the acquisition of novel invertase activities. A classic view popularized by Ohno [78], is that gene family expansions associated with gene duplications are the principal source of new genes that acquire new functions. This is because duplication creates a redundant gene copy that is free from selection and that can evolve a new function (i.e., neofunctionalization). It is therefore possible that following the gene duplication, relaxed selection allowed for the acquisition of novel domains by the GH32 paralogs. During this process, one of the *Ceratocystis* paralogs likely acquired the transmembrane region characteristic of vacuolar invertases, while the other acquired the eukaryotic signal motif characteristic of cell wall invertases. Based on the results of our ML and CAFE analyses, the evolution of the *Huntiella* GH32 gene followed a parallel evolutionary trajectory during which it independently acquired its eukaryotic signal motif.

As have been demonstrated for other Ascomycetes [2], the data presented here suggested a link between the ecological strategy of Ceratocystidaceae and GH32 gene family size. In fungi, changes in the repertoire of GH32 functional products are thought to influence the efficiency at which sucrolytic compounds are exploited [79]. In the Hypocreales, for example, the respective GH32 family expansions and contractions appear to be linked to the evolution of the Nectriaceae with their plant pathogenic lifestyles [80], and to that of the Cordycipitaceae-Clavicipitaceae clade that are often insect pathogens or have undergone a host jump from insects to plants [81]. The evolution of the Glomeralles also appeared to be associated with such changes in the GH32 family, where a significant contraction was observed at the base of the Plectosphaerellaceae with its alkaliphilic representatives [82], while the Glomerellaceae clade with its plant pathogens [83] were associated with several significant expansions. Plant associated fungi likely adapted to hosts through a larger repertoire of invertases that allow these species to access plant-synthesised sucrose [2]. This might be the case for *Ceratocystis* species with their two GH32 invertases. On the other hand, restrictions in functional invertase repertoires (e.g., in the saprophytic *Huntiella*) might be important for exploiting niches with limited sucrose resources, as well as for potentially avoiding plant defence mechanisms, thus conferring the ability to colonise plant-associated niches [84]. Although the apparent link between GH32 gene family size and the ecology of the Ceratocystidaceae is consistent with the results of previous studies [2, 75], additional work is needed to fully understand the role(s) of GHs or carbohydrases available to these fungi in determining their ecological capabilities.

Similar to previous studies, results of this study suggest that transposon-like elements may have played a role in the evolution of the Ceratocystidaceae GH32 invertases. For example, retrotransposon-like elements that are part of Class I transposable elements (TEs) [5] have been used to explain why the number of introns differ between certain groups of plant invertases [5]. Local synteny information and intron conservation ratios indicated that the *Huntiella* invertase might represent a retrotransposed copy of the ancestral gene (i.e., the ancestral GH32 gene that gave rise to all of the Ceratocystidaceae genes examined here). Similar to what has been shown for other retrotransposed gene copies [43], the *Huntiella* invertase genes lack introns, and the genomic region containing them appears to be non-homologous to the invertase gene-bearing genomic region of *Ceratocystis* (i.e., the GH32 genes of these two genera are flanked by completely different sets of genes). Retrotransposons facilitate intron loss/gain via a copy and paste mechanism involving, first, reverse transcription of messenger RNA (mRNA) into complementary DNA (cDNA), followed by homologous recombination between the original gene (or a homolog) and cDNA [55]. Therefore, as have been suggested for *Oryza sativa* and *A. thaliana* [5], the activity of retrotransposon-like elements in the genomes of the Ceratocystidaceae and its ancestors could have been responsible for or involved in the initial loss of one of the two ancestral GH32 genes predicted for the Ceratocystidaceae, and the subsequent duplication in the LCA of *Ceratocystis*.

Another group of transposon-like elements that could have influenced the evolution of the Ceratocystidaceae invertases is the *Fot5* or pogo-like elements (Class II of TEs; also referred to as DNA transposons). *Fot5* utilizes a ‘cut-and-paste’ mechanism for transpositioning, during which a specific DNA region is excised and inserted into a target site elsewhere in the genome [85]. The activity of *Fot5* in *Ceratocystis* may thus have given rise to genomic rearrangements that also affected the region harbouring the two GH32 invertase genes. In fact, the apparent abundance of *Fot5* homologs in the genomes of the *Ceratocystis* species and the presence of short terminal branches on the *Fot5* phylogeny suggests that these elements were active relatively recently [56]. Our *Fot5* phylogeny further suggests that many *Fot5* elements were active in the ancestral lineages of *Ceratocystis* (i.e., homologs from different *Ceratocystis* species group together in a cluster), while others were active after speciation (i.e., homologs represent unique *Fot5* lineages or group according to species) [56]. Analysis of the *Ceratocystis Fot5* elements also showed that their lifestyles most likely match those of other TEs and parasitic DNA elements [85]. Once inside the genome of the fungal individual, the *Fot5* element likely increased in copy number and persisted until all its copies become inactive due to either vertical inactivation by the TE itself [86] or host-associated mechanisms that protect the genome from parasitic DNA elements (e.g., RIP) [55, 85]. Indeed, our analysis of the *Fot5* elements suggested a possible RIP response in *Ceratocystis*. Over time, these inactivated copies will degenerate further through mutation and genetic drift, until no identifiable remnants of the original TE remain in the genome [85]. The fact that none of the three *Huntiella* genomes harboured detectable *Fot5* elements thus suggests that the lineage never harboured these TEs, and if they were present they have degenerated to a point where standard *in silico* tools can no longer detect them.

An important hypothesis emerging from this study is that the activity of *Fot5* elements facilitated assembly of a genomic region or island key to the ecological success of *Ceratocystis* species. In addition to the two GH32 invertase genes, this genomic region encodes various other genes potentially involved in the ability of this taxon to infect and colonize health woody and herbaceous plants. In *Fusarium*, the genomic regions harbouring *Fot5* elements are commonly associated with strain- or species-specific regions that are enriched for genes involved in pathogenicity and/or adaptation [87]. Virulence genes in other pathogens are also often found in genomic regions dense with TEs where the genomic plasticity associated with these elements is believed to contribute to the evolution of virulence and pathogenicity related genes [88]. The GH32-bearing genomic region identified in *Ceratocystis* may therefore represent a key target for future studies into the molecular basis of the ability of these fungi to cause plant disease. Also, further investigation of the diversity and evolution of *Fot5* and other TEs will undoubtedly provide valuable clues regarding gene and genome evolution in the Ceratocystidaceae with their diverse ecologies, modes of reproduction and potential biotechnological benefits.

## Conclusions

In this study, we considered the capacity of Ceratocystidaceae and a selection of Sordariomycetes species to utilize sucrose by GH32 invertase enzymes. The publicly available genome sequences for these taxa, and the *H. savannae* genome sequenced here, were used to identify novel GH32-like sequences. The number of GH32 gene family members in a particular fungus appeared to be related to the ecological strategy employed by the fungus, which was similar to previous studies. The genomes of the plant pathogenic *Ceratocystis* species harboured two invertase genes. This was in contrast to their saprophytic relatives in the genus *Huntiella* that contained only one. Our results further showed that several processes have shaped the evolutionary trajectories of these Ceratocystidaceae genes. Based on these data, we posit that the evolution of the Ceratocystidaceae GH32 gene family involved divergence of invertase gene paralogs that presumably arose from a single Group 8 type of intracellular invertases present in the LCA of this fungal family. These paralogs acquired specific terminal motifs to give rise to genes encoding a cell wall invertase and a vacuolar invertase in extant species of *Ceratocystis*. A similar scenario likely also occurred in *Huntiella* where the ancestral invertase was remodelled into a cell wall invertase through the acquisition of relevant sequence motifs. The genes in the GH32 family of *Ceratocystis* and *Huntiella* were also located at non-homologous loci or regions in the genomes and were flanked by completely different sets of genes in the examined species, which indicated these genes are not orthologous (*sensu* Koonin; [36]) between the two sister genera. The genomic rearrangement that caused this was potentially linked to the activity of the putative *Fot5* element(s) found in *Ceratocystis*. Our results thus suggested a role for TEs in shaping the evolution of GH32 family genes, and thereby the sucrolytic activities and related ecological strategies of the Ceratocystidaceae that harbour them.

### Availability of supporting data

This Whole Genome Shotgun project has been deposited at DDBJ/EMBL/GenBank under the accession LCZG00000000. The version described in this paper is version LCZG01000000.

## Additional files

Additional file 1: Table S1. Genomic location, Protein ID and GenBank accession numbers for the sequences used in the present study. (DOC 67 kb) Additional file 2: Figure S1. The chronogram was inferred using published calibration time points (see text for detail) and a Bayesian strict clock approach as implemented in BEAST (Bayesian evolutionary analysis by sampling trees) v.2 package v.2.2.1 [46]. Horizontal bars mark the lower and upper time boundaries of the indicated mean age (Million years ago) estimates of the nodes. The black arrows indicate the four calibration points, which include the Dothideomycetes crown group (mean 350 Million years ago [Mya] with 95 % credibility interval [CI] of 273–459) [50], the last common ancestor (LCA) of the Hypocreales (181 Mya with 95 % CI of 150–213) [51], the Clavicipitaceae crown group (117 Mya with 95 % CI of 95–144) [51], as well the Nectriaceae crown group (125 Mya with 95 % CI of 98–155) [52]. (PDF 227 kb) Additional file 3: Table S2. List of the putative *Fot5* homologs identified in this study. (DOC 160 kb) Additional file 4: Figure S2. Maximum likelihood tree of the *Fot5* DDD catalytic domain. This analysis was done using the WAG substitution model [49] and gamma correction to account for among site rate variation. Percentage bootstrap support (based on a 1000 repeats, with cut-off value of 50 %) is indicated at the internodes. Genomic coordinates of putative *Ceratocystis Fot5* homologs are provided in Additional file 3: Table S2. GenBank accession numbers or sequence identifiers for previously identified *Fot5* homologs are: Fot2 [Genbank:JN624854, *F. oxysporum*), *Fot5* [Genbank:CAE55867, *F. oxysporum*], Fot1 [Genbank:X64799, *F. oxysporum*], Fot4 [Genbank:AF076632, *F. oxysporum*], Fot9 [JGI:2517, *F. graminearum*], Fotyl [Genbank:CAG33729.1 *Yarrowia lipolytica*], Molly [Genbank:CAD32687, *Parastagonospora nodorum*], Ophio [Genbank:ABG26269, *Ophiostoma novo-ulmi*], PABRA [Genbank:ACY56713, *Paracoccidioides brasiliensis*], Pixie [Genbank:CAD32689, *Parastagonospora nodorum*], Pot2 [Genbank:CAA83918, *Magnaporthe grisea*], Pot3 [Genbank:AAC49418, *M. grisea*], SCSCL [Genbank:XP001592252, *Sclerotinia sclerotiorum*], Taf1 [Genbank:AAX83011, *Aspergillus fumigatus*], Tan1 [Genbank:U58946, *Aspergillus awamori*] USMA [Genbank:UM03882, *Ustilago maydis*), Flipper [Genbank:AAB63315, *Botryotinia fuckeliana*] and Cirt1 [Genbank:XP710204, *Candida albicans*]. For the *Ceratocystis* sequences JS = *Ceratocystis albifundus* (green dots), J = *Ceratocystis manginecans* (red dots) and A = *Ceratocystis fimbriata* (blue dots), followed by the genomic position. (PDF 59 kb)

## References

[1] Alberto F, Bignon C, Sulzenbacher G, Henrissat B, Czjzek M (2004). The three-dimensional structure of invertase (β-fructosidase) from Thermotoga maritima reveals a bimodular arrangement and an evolutionary relationship between retaining and inverting glycosidases. J Biol Chem.

[2] Parrent JL, James TY, Vasaitis R, Taylor AF (2009). Friend or foe? Evolutionary history of glycoside hydrolase family 32 genes encoding for sucrolytic activity in fungi and its implications for plant-fungal symbioses. BMC Evol Biol.

[3] Lammens W, Le Roy K, Schroeven L, Van Laere A, Rabijns A, Van den Ende W (2009). Structural insights into glycoside hydrolase family 32 and 68 enzymes: functional implications. J Exp Bot.

[4] Bocock PN, Morse AM, Dervinis C, Davis JM (2008). Evolution and diversity of invertase genes in *Populus trichocarpa*. Planta.

[5] Ji X, van den Ende W, van Laere A, Cheng S, Bennett J (2005). Structure, evolution, and expression of the two invertase gene families of rice. J Mol Evol.

[6] Cantarel BLCP, Rancurel C, Bernard T, Lombard V, Henrissat B (2009). The Carbohydrate-active enzymes database (CAZY): an expert resource for glycogenomics. Nucleic Acids Res.

[7] Naumoff DG (2001). Beta-fructosidase superfamily: homology with some alpha-l-arabinases and beta-d-xylosidases. Proteins.

[8] Naumoff DG (2012). Furanosidase superfamily: search of homologues. Mol Biol.

[9] Álvaro-Benito M, Polo A, González B, Fernández-Lobato M, Sanz-Aparicio J (2010). Structural and kinetic analysis of Schwanniomyces occidentalis invertase reveals a new oligomerization pattern and the role of its supplementary domain in substrate binding. J Biol Chem.

[10] Lombard V, Ramulu HG, Drula E, Coutinho PM, Henrissat B (2014). The carbohydrate-active enzymes database (CAZy) in 2013. Nucleic Acids Res.

[11] Roitsch T, González MC (2004). Function and regulation of plant invertases: sweet sensations. Trends Plant Sci.

[12] Tang G-Q, Lüscher M, Sturma A (1999). Antisense repression of vacuolar and cell wall invertase in transgenic carrot alters early plant development and sucrose partitioning. Plant Cell.

[13] Sharma R, Cao P, Jung K-H, Sharma MK, Ronald PC. Construction of a rice glycoside hydrolase phylogenomic database and identification of targets for biofuel research. Front Plant Sci. 2013;4:330. 10.3389/fpls.2013.00330 PMC3752443 23986771

[14] Maiorano AE, Piccoli RM, Da Silva ES, De Andrade Rodrigues MF (2008). Microbial production of fructosyltransferases for synthesis of pre-biotics. Biotechnol Lett.

[15] Nadeem H, Rashid MH, Siddique MH, Azeem F, Muzammil S, Javed MR, et al. Microbial invertases: A review on kinetics, thermodynamics, physiochemical properties. Process Biochemistry 2015, doi:10.1016/j.procbio.2015.04.015.

[16] Aguiar TQ, Dinis C, Magalhães F, Oliveira C, Wiebe MG, Penttilä M, Domingues L (2014). Molecular and functional characterization of an invertase secreted by *Ashbya gossypii*. Mol Biotechnol.

[17] Carlson M, Botstein D (1982). Two differentially regulated mRNAs with different 50 ends encode secreted and intracellular forms of yeast invertase. Cell.

[18] Nafisi M, Stranne M, Zhang L, van Kan JA, Sakuragi Y (2014). The endo-arabinanase BcAra1 is a novel host-specific virulence factor of the necrotic fungal phytopathogen *Botrytis cinerea*. Mol Plant Microbe Interact.

[19] Wang Y, Wang X, Tang H, Tan X, Ficklin SP, Feltus FA, Paterson AH (2011). Modes of gene duplication contribute differently to genetic novelty and redundancy, but show parallels across divergent Angiosperms. PLoS One.

[20] de Beer ZW, Duong TA, Barnes I, Wingfield BD, Wingfield MJ (2014). Redefining *Ceratocystis* and allied genera. Stud Mycol.

[21] Wilken PM, Steenkamp ET, Wingfield MJ, De Beer ZW, Wingfield BD (2013). *Ceratocystis fimbriata*: draft nuclear genome sequence for the plant pathogen, *Ceratocystis fimbriata*. IMA Fungus.

[22] Wingfield BD, van Wyk M, Roos H, Wingfield MJ, Seifert KA, de Beer ZW, Wingfield MJ (2013). *Ceratocystis*: Emerging evidence for discrete generic boundaries. The Ophiostomatoid fungi: Expanding frontiers.

[23] Baker CJ, Harrington TC, Krauss U, Alfenas AC (2003). Genetic variability and host specialization in the Latin American clade of *Ceratocystis fimbriata*. Phytopathology.

[24] Van Wyk M, Adawi AOA, Khan IA, Deadman ML, Al Jahwari AA, Wingfield BD, Ploetz R, Wingfield MJ (2007). *Ceratocystis manginecans* sp. nov, causal agent of a destructive mango wilt disease in Oman and Pakistan. Fungal Divers.

[25] Roux J, Meke G, Kanyi B, Mwangi L, Mbaga A, Hunter GC, Nakabonge G, Heath RN, Wingfield MJ (2005). Diseases of plan tation forestrytrees in eastern and Southern Africa. S Afr J Sci.

[26] Van der Nest MA, Bihon W, De Vos L, Naidoo K, Roodt D, Rubagotti E, Slippers B, Steenkamp ET, Wilken PM, Wilson A (2014). Draft genome sequences of *Diplodia sapinea*, *Ceratocystis manginecans*, and *Ceratocystis moniliformis*. IMA Fungus.

[27] Van der Nest MA, Beirn LA, Crouch JA, Demers JE, De Beer ZW, De Vos L, Gordon TR (2014). Draft genomes of *Amanita jacksonii, Ceratocystis albifundus, Fusarium circinatum, Huntiella omanensis, Leptographium procerum, Rutstroemia sydowiana*, and *Sclerotinia echinophila*. IMA Fungus.

[28] Van Wyk N, Trollope KM, Steenkamp ET, Wingfield BD, Volschenk H (2013). Identification of the gene for beta-fructofuranosidase from *Ceratocystis moniliformis* CMW 10134 and characterization of the enzyme expressed in *Saccharomyces cerevisiae*. BMC Biotechnol.

[29] Chen S, Van Wyk M, Roux J, Wingfield MJ, Xie Y, Zhou X (2013). Taxonomy and pathogenicity of *Ceratocystis* species on Eucalyptus trees in South China, including *C. chinaeucensis* sp. nov. Fungal Divers.

[30] Barnes I, Gaur A, Burgess T, Roux J, Wingfield BD, Wingfield MJ (2001). Microsatellite markers reflect intra‐specific relationships between isolates of the vascular wilt pathogen *Ceratocystis fimbriata*. Mol Plant Pathol.

[31] Boetzer M, Henkel CV, Jansen HJ, Butler D, Pirovano W (2011). Scaffolding pre-assembled contigs using SSPACE. Bioinformatics.

[32] Stanke M, Diekhans M, Baertsch R, Haussler D (2008). Using native and syntenically mapped cdna alignments to improve de novo gene finding. Bioinformatics.

[33] Parra G, Bradnam K, Korf I (2007). CEGMA: a pipeline to accurately annotate core genes in eukaryotic genomes. Bioinformatics.

[34] Gabaldón T. Large-scale assignment of orthology: back to phylogenetics. Genome Biol. 2008;9:235. 10.1186/gb-2008-9-10-235 PMC2760865 18983710

[35] Li L, Stoeckert C, Roos D (2003). OrthoMCL: identification of ortholog groups for eukaryotic genomes. Genome Res.

[36] Koonin E (2005). Orthologs, paralogs, and evolutionary genomics. Annu Rev Genet.

[37] Gupta S, Singh M (2015). Phylogenetic method for high-throughput ortholog detection. Inform Eng Electron Bus.

[38] Katoh K, Standley DM (2013). Mafft multiple sequence alignment software version 7: improvements in performance and usability. Mol Biol Evol.

[39] Guindon S, Dufayard J-F, Lefort V, Anisimova M, Gascuel O (2010). New algorithms and methods to estimate maximum-likelihood phylogenies: assessing the performance of PHYML 3.0. Syst Biol.

[40] Abascal F, Zardoya R, Posaa D (2005). ProtTest: selection of best-fit models of protein evolution. Bioinformatics.

[41] Le SQ, Gascuel O (2008). An improved general amino acid replacement matrix. Mol Biol Evol.

[42] Tamura K, Stecher G, Peterson D, Filipski A, Kumar S (2013). Mega6: molecular evolutionary genetics analysis version 6.0. Mol Biol Evol.

[43] Jun J, Mandoiu II, Nelson CE. Identification of mammalian orthologs using local synteny. BMC Genomics. 2009;10:630. 10.1186/1471-2164-10-630 PMC2807883 20030836

[44] Conesa A, Götz S, García-Gómez J, Terol J, Talón M, Robles M (2005). Blast2GO: a universal tool for annotation, visualization and analysis in functional genomics research. Bioinformatics.

[45] Han MV, Thomas GW, Lugo-Martinez J, Hahn MW (2013). Estimating gene gain and loss rates in the presence of error in genome assembly and annotation using CAFE 3. Mol Biol Evol.

[46] Drummond A, Suchard MA, Xie D, Rambaut A (2012). Bayesian phylogenetics with BEAUti and the BEAST 1.7. Mol Biol Evol.

[47] Schoch CL, Seifert KA, Huhndorf S, Robert V, Spouge JL, Levesque CA, Chen W, Consortium FB (2012). Nuclear ribosomal internal transcribed spacer (ITS) region as a universal DNA barcode marker for Fungi. Proc Natl Acad Sci.

[48] Stielow J, Lévesque C, Seifert K, Meyer W, Irinyi L, Smits D, Renfurm R, Verkley G, Groenewald M, Chaduli D (2015). One fungus, which genes? Development and assessment of universal primers for potential secondary fungal DNA barcodes.

[49] Whelan S, Goldman N (2001). A general empirical model of protein evolution derived from multiple protein families using a maximum-likelihood approach. Mol Biol Evol.

[50] Prieto M, Wedin M (2013). Dating the diversification of the major lineages of Ascomycota (Fungi). PLoS One.

[51] Yang E, Lingling X, Ying Y, Xinyu Z, Meichun X, Chengshu W, Zhiqiang A, Xingzhong L (2012). Origin and evolution of carnivorism in the Ascomycota (fungi). Proc Natl Acad Sci.

[52] Sung GH, Poinar GO, Spatafora JW (2008). The oldest fossil evidence of animal parasitism by fungi supports a Cretaceous diversification of fungal–arthropod symbioses. Mol Phylogenet Evol.

[53] Yule GU (1924). A mathematical theory of evolution, based on the conclusions of Dr. JC Willis, FRS. Philosophical Transactions of the Royal Society of London.

[54] Rep M, Van Der Does HC, Meijer M, Van Wijk R, Houterman PM, Dekker HL, Cornelissen BJ (2004). A small, cysteine‐rich protein secreted by *Fusarium oxysporum* during colonization of xylem vessels is required for I‐3‐mediated resistance in tomato. Mol Microbiol.

[55] Daboussi M-J, Capy P (2003). Transposable elements in filamentous fungi. Annu Rev Microbiol.

[56] Dufresne M, Lespinet O, Daboussi M-J, Hua-Van A (2011). Genome-wide comparative analysis of pogo-like transposable elements in different Fusarium species. J Mol Evol.

[57] Galagan J, Selker E (2004). RIP: the evolutionary cost of genome defense. Trends Genet.

[58] Hane J, Oliver R (2008). RIPCAL: a tool for alignment-based analysis of repeat-induced point mutations in fungal genomic sequences. BMC Bioinformatics.

[59] Altenbach D, Nüesch E, Meyer AD, Boller T, Wiemken A (2004). The large subunit determines catalytic specificity of barley sucrose:fructan 6-fructosyltransferase and fescue sucrose:sucrose 1-fructosyltransferase. FEBS Lett.

[60] Reddy A, Maley F (1996). Studies on identifying the catalytic role of glu-204 in the active site of yeast invertase. J Biol Chem.

[61] Pagny S, Denmat-Ouisse LA, Gomord V, Faye L (2003). Fusion with HDEL protects cell wall invertase from early degradation when N-glycosylation is inhibited. Plant Cell Physiol.

[62] Tauzin AS, Giardina T (2014). Sucrose and invertases, a part of the plant defense response to the biotic stresses. Front Plant Sci.

[63] Goosen C, Yuan XL, van Munster JM, Ram AF, van der Maarel MJ, Dijkhuizen L (2007). Molecular and biochemical characterization of a novel intracellular invertase from *Aspergillus niger* with transfructosylating activity. Eukaryot Cell.

[64] Moriyama S, Tanaka H, Uwataki M, Muguruma M, Ohta K (2003). Molecular cloning and characterization of an exoinulinase gene from *Aspergillus niger* Strain 12 and its expression in *Pichia pastoris*. J Biosci Bioeng.

[65] Beimforde C, Feldberg K, Nylinder S, Rikkinen J, Tuovila H, Dörfelt H, Gube M, Jackson DJ, Reitner J, Seyfullah LJ (2014). Estimating the Phanerozoic history of the Ascomycota lineages: combining fossil and molecular data. Mol Phylogenet Evol.

[66] Schroeven L, Lammens W, Van Laere A, Van den Ende W (2008). Transforming wheat vacuolar invertase into a high affinity sucrose:sucrose 1-fructosyltransferase. New Phytol.

[67] Ruan Y-L (2014). Sucrose metabolism: gateway to diverse carbon use and sugar signaling. Annu Rev Plant Biol.

[68] Kulshrestha S, Tyagi P, Sindhi V, Yadavilli KS (2013). Invertase and its applications–a brief review. J Pharm Res.

[69] Sun L, Yang D, Kong Y, Chen Y, Li XZ, Zeng LJ, Li Q, Wang ET, He ZH (2013). Sugar homeostasis mediated by cell wall invertase GRAIN INCOMPLETE FILLING 1 (GIF1) plays a role in pre-existing and induced defence in rice. Mol Plant Pathol.

[70] Tetlow IJ, Farrar JF (1992). Sucrose-metabolizing enzymes from leaves of barley infected with brown rust (*Puccinia hordeiotth*). New Phytol.

[71] Voegele RT, Stefan W, Ulla M, Melanie L, Kurt M (2006). Cloning and characterization of a novel invertase from the obligate biotroph Uromyces fabae and analysis of expression patterns of host and pathogen invertases in the course of infection. Mol Plant Microbe Interact.

[72] Hayes MA, Feechan A, Dry IB (2010). Involvement of abscisic acid in the coordinated regulation of a stress-inducible hexose transporter (VvHT5) and a cell wall invertase in grapevine in response to biotrophic fungal infection. Plant Physiol.

[73] Schirawski J (2015). Invasion is sweet. New Phytol.

[74] Fridman E, Zamir D (2003). Functional divergence of a syntenic invertase gene family in tomato, potato, and arabidopsis. Plant Physiol.

[75] Naumoff DG (2011). Hierarchical classification of glycoside hydrolases. Biochemistry.

[76] Yao Y, Meng-Ting G, Xiao-Hui W, Jiao L, Rui-Mei L, Xin-Wen H, Jian-Chun G (2014). Genome-wide identification, 3D modeling, expression and enzymatic activity analysis of cell wall invertase gene family from cassava (*Manihot esculenta* Crantz). Int J Mol Sci.

[77] Hahn MW, De Bie T, Stajich JE, Nguyen CN, Cristianini N (2005). Estimating the tempo and mode of gene family evolution from comparative genomic data. Genome Res.

[78] Ohno S (1970). Evolution by gene duplication.

[79] Bergthorsson U, Andersson D, Roth J (2007). Ohno’s dilemma: evolution of new genes under continuous selection. Proc Natl Acad Sci.

[80] Goswami RS, Kistler HC (2004). Heading for disaster: *Fusarium graminearum* on cereal crops. Mol Plant Pathol.

[81] Spatafora JW, Sung GH, Sung JM, HYWEL‐JONES NL, White JF (2007). Phylogenetic evidence for an animal pathogen origin of ergot and the grass endophytes. Mol Ecol.

[82] Grum-Grzhimaylo AA, Debets AJM, van Diepeningen AD, Georgieva ML, Bilanenko EN (2013). *Sodiomyces alkalinus*, a new holomorphic alkaliphilic ascomycete within the Plectosphaerellaceae. Persoonia.

[83] Hyde KD, Jones EBG, Liu J-K, Ariyawansa H, Boehm E, Boonmee S, Braun U, Chomnunti P, Crous PW, Dai D-Q (2013). Families of Dothideomycetes. Fungal Divers.

[84] Aguileta G, Hood ME, Refregier G, Giraud T (2009). Genome evolution in plant pathogenic and symbiotic fungi. Adv Bot Res.

[85] Munoz-Lopez M, Garcia-Perez JL (2010). DNA transposons: nature and applications in genomics. Curr Genomics.

[86] Lohe A, Moriyama E, Lidholm D, Hartl D (1995). Horizontal transmission, vertical inactivation, and stochastic loss of mariner -like transposable elements. Mol Biol Evol.

[87] MA LJ, Van Der Does HC, Borkovich KA, Coleman JJ, Daboussi MJ, Di Pietro A (2010). Comparative genomics reveals mobile pathogenicity chromosomes in *Fusarium*. Nature.

[88] Thon MR, Pan H, Diener S, Papalas J, Taro A, Mitchell TK, Dean RA (2006). The role of transposable element clusters in genome evolution and loss of synteny in the rice blast fungus *Magnaporthe oryzae*. Genome Biol.

[89] Chen K, Durand D, Farach-Colton M (2000). Notung: a program for dating gene duplications and optimizing gene family trees. J Comput Biol.

