# Supplementary material for: Saprophytic and pathogenic fungi in the Ceratocystidaceae differ in their ability to metabolize plant-derived sucrose
Source: BMC Evol Biol. 2015 Dec 7;15:273. doi: 10.1186/s12862-015-0550-7 (PMC4672557; doi:10.1186/s12862-015-0550-7)
Supplement: Additional file 1: Table S1. — Genomic location, Protein ID and GenBank accession numbers for the sequences used in the present study. (DOC 67 kb) [file 12862_2015_550_MOESM1_ESM.doc]

**Additional file 1: Table S1** Genomic location, Protein ID and GenBank accession numbers for the sequences used in the present study.

| **Taxon** | **RPB2** | **RPB1** | **MCM7** | **EF3** | **EF1** |
| --- | --- | --- | --- | --- | --- |
| *Acremonium alcalophilum* v2.0 | 1062944 | 1093047 | 2025206 | 2131370 | 1054920 |
| *Alternaria brassicicola* | 3555 | 6953 | 8151 | 6101 | 4028 |
| *Anthostoma avocetta* NRRL 3190 v1.0 | 358799 | 486375 | 366025 | 358521 | 427765 |
| *Apiospora montagnei* NRRL 25634 v1.0 | 165778 | 493678 | 198198 | 50284 | 53056 |
| *Beauveria bassiana* ARSEF 2860 | 10291 | 260 | 1172 | 6276 | 2388 |
| *Ceratocystis albifundus* | JSSU01001074: 17650-16520 | JSSU01001372: 98713-99534 | JSSU01000973: 6705-7463 | JSSU01001081: 14425-15222 | JSSU01001043: 14465-13434 |
| *Ceratocystis fimbriata* | APWK02000915: 18679-19938 | APWK02000296: 6011-5382 | KM495432 | APWK02000274: 34298-33501 | APWK02000140: 31660-32691 |
| *Ceratocystis manginecans* | JJRZ01000317: 13970-12711 | JJRZ01000001: 51511-50882 | KM495440 | JJRZ01000106: 53401-52604 | JJRZ01000044: 8654-9685 |
| *Chaetomium globosum* v1.0 | 18331 | 11528 | 10900 | 14798 | 16368 |
| *Colletotrichum graminicola* M1.001 | 719 | 3226 | 9817 | 7890 | 8402 |
| *Colletotrichum higginsianum* IMI 349063 | 11479 | 3226 | 4433 | 9852 | 13479 |
| *Coniochaeta ligniaria* NRRL30616 V.1.0 | 10961 | 2621 | 5349 | 5900 | 10615 |
| *Cordyceps militaris* CM01 | 6678 | 5469 | 5557 | 5648 | 808 |
| *Cryphonectria parasitica* EP155 v2.0 | 348358 | 81184 | 263526 | 254675 | 355033 |
| *Daldinia eschscholzii* EC12 v1.0 | 318358 | 53585 | 385894 | 386825 | 320720 |
| *Eutypa lata* UCREL1 | 2978 | 7447 | 9192 | 4830 | 6820 |
| *Fusarium fujikuroi* IMI 58289 | 7812 | 1359 | 4389 | 12267 | 8475 |
| *Fusarium graminearum* v1.0 | 3039 | 1079 | 8215 | 3861 | 9400 |
| *Fusarium oxysporum v1.0* | 3867 | 884 | 7088 | 4679 | 11183 |
| *Fusarium verticillioides 7600* v1.0 | 3039 | 685 | 1814 | 4125 | 7139 |
| *Glomerella acutata v1.0* | 1356853 | 1444979 | 1457852 | 1571219 | 1333254 |
| *Glomerella cingulata 23 v1.0* | 1845705 | 1727691 | 1819621 | 1725208 | 1771438 |
| *Grosmannia* *clavigera* kw1407 | 3040 | 7178 | 6801 | 2788 | 3764 |
| *Huntiella moniliformis* | JMSH01000100: 35095-33929 | JMSH01000104: 27732-28352 | JMSH01000011: 212164-211406 | JMSH01000009: 328536-329333 | JMSH01000070: 123062-124093 |
| *Huntiella omanensis* | JSUI01006186: 9662-8514 | JSUI01006274: 17350-16709 | JSUI01006482: 39381-40139 | JSUI01006507: 26080-25283 | JSUI01006383: 43877-44908 |
| *Huntiella savannae* | NODE_6005: 96370-95237 | NODE_3982: 106186-105545 | KM495462 | NODE_4668: 115552-114755 | NODE_3974: 9462-8431 |
| *Hypoxylon* sp. CI-4A v1.0 | 11137 | 34528 | 1393894 | 123171 | 1390744 |
| *Ilyonectria* sp. v1.0 | 1466688 | 1624773 | 1550485 | 1535645 | 1528939 |
| *Metarhizium acridum* CQMa 102 | 2463 | 7408 | 1075 | 4249 | 964 |
| *Metarhizium robertsii* ARSEF 23 | 336 | 658 | 5659 | 2762 | 3797 |
| *Myceliophthora thermophila* v2.0 | 2307570 | 2294525 | 2295768 | 73461 | 2298136 |
| *Mycosphaerella_fijiensise_*v2.0 | 136855 | 58269 | 56719 | 210369 | 209593 |
| *Nectria haematococca v2.0* | 70495 | 103209 | 92685 | 70096 | 59329 |
| *Neurospora crassa* OR74A v2.0 | 9487 | 328 | 89 | 5493 | 436 |
| *Neurospora discreta* FGSC 8579 mat A | 128789 | 73118 | 91067 | 166279 | 160296 |
| *Neurospora tetrasperma* FGSC 2508 mat A v2.0 | 124967 | 70543 | 88816 | 116274 | 118521 |
| Ophiostoma piceae UAMH 11346 | 2689 | 816 | 8019 | 1044 | 3764 |
| *Phaeoacremonium aleophilum* UCRPA7 | 5318 | 5500 | 7516 | 4179 | 4720 |
| *Podospora anserina* S mat+ | 5635 | 297 | 726 | 4339 | 2076 |
| *Sodiomyces alkalinus* v1.0 | 273095 | 143113 | 334328 | 354039 | 318117 |
| *Stagonospora_nodorum* *SN15 v2.0* | 4533 | 4606 | 10072 | 11732 | 5231 |
| *Thielavia antarctica* CBS 123565 v1.0 | 442445 | 184819 | 85725 | 445493 | 442817 |
| *Thielavia appendiculata* CBS 731.68 v1.0 | 581449 | 562737 | 639459 | 647848 | 350199 |
| *Thielavia arenaria* CBS 508.74 v1.0 | 783437 | 700748 | 774767 | 778860 | 773701 |
| *Thielavia hyrcaniae* CBS 757.83 v1.0 | 499486 | 128871 | 556260 | 499996 | 494733 |
| *Thielavia terrestris* v2.0 | 2110960 | 2121340 | 2114018 | 2115984 | 2111611 |
| *Trichoderma asperellum* CBS 433.97 v1.0 | 77086 | 68995 | 157112 | 35803 | 158395 |
| *Trichoderma atroviride* V2.0 | 151043 | 89498 | 294914 | 156340 | 300828 |
| *Trichoderma harzianum* CBS 226.95 v1.0 | 9591 | 508530 | 490345 | 518654 | 12328 |
| *Trichoderma longibrachiatum* ATCC 18648 v3.0 | 58810 | 67760 | 1427374 | 1398382 | 1397263 |
| *Trichoderma reesei* v2.0 | 79225 | 79315 | 3212 | 71363 | 46958 |
| *Trichoderma virens* Gv29-8 v2.0 | 76818 | 85034 | 74376 | 74376 | 83874 |
| *Verticillium dahliae* v1.0 | 1143 | 1942 | 9482 | 3649 | 7717 |
