# Supplementary figures and images for: Saprophytic and pathogenic fungi in the Ceratocystidaceae differ in their ability to metabolize plant-derived sucrose
Source: BMC Evol Biol. 2015 Dec 7;15:273. doi: 10.1186/s12862-015-0550-7 (PMC4672557; doi:10.1186/s12862-015-0550-7)

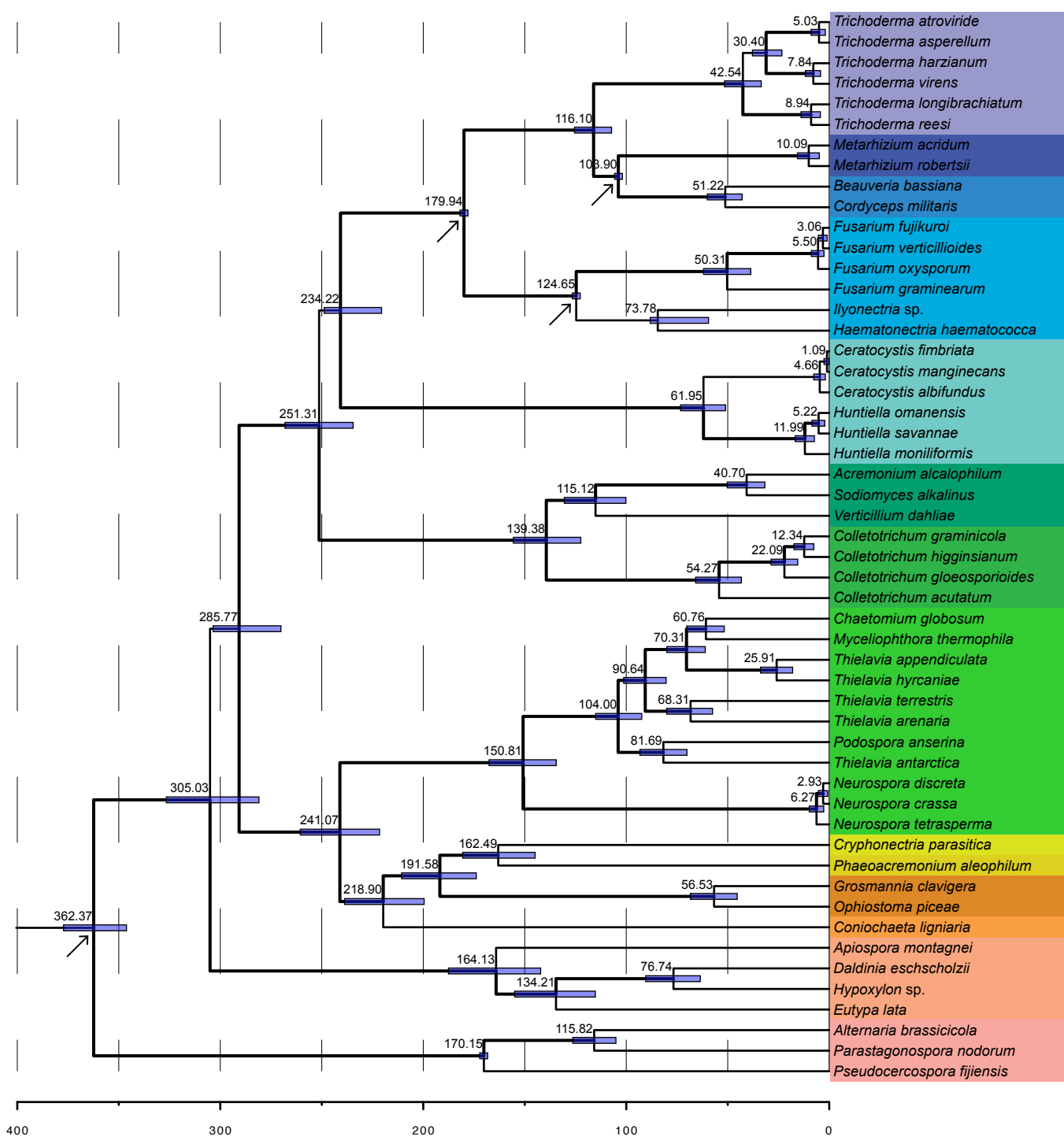

Supplement: Additional file 2: Figure S1. — The chronogram was inferred using published calibration time points (see text for detail) and a Bayesian strict clock approach as implemented in BEAST (Bayesian evolutionary analysis by sampling trees) v.2 package v.2.2.1 [46]. Horizontal bars mark the lower and upper time boundaries of the indicated mean age (Million years ago) estimates of the nodes. The black arrows indicate the four calibration points, which include the Dothideomycetes crown group (mean 350 Million years ago [Mya] with 95 % credibility interval [CI] of 273–459) [50], the last common ancestor (LCA) of the Hypocreales (181 Mya with 95 % CI of 150–213) [51], the Clavicipitaceae crown group (117 Mya with 95 % CI of 95–144) [51], as well the Nectriaceae crown group (125 Mya with 95 % CI of 98–155) [52]. (PDF 227 kb) [file 12862_2015_550_MOESM2_ESM.pdf]

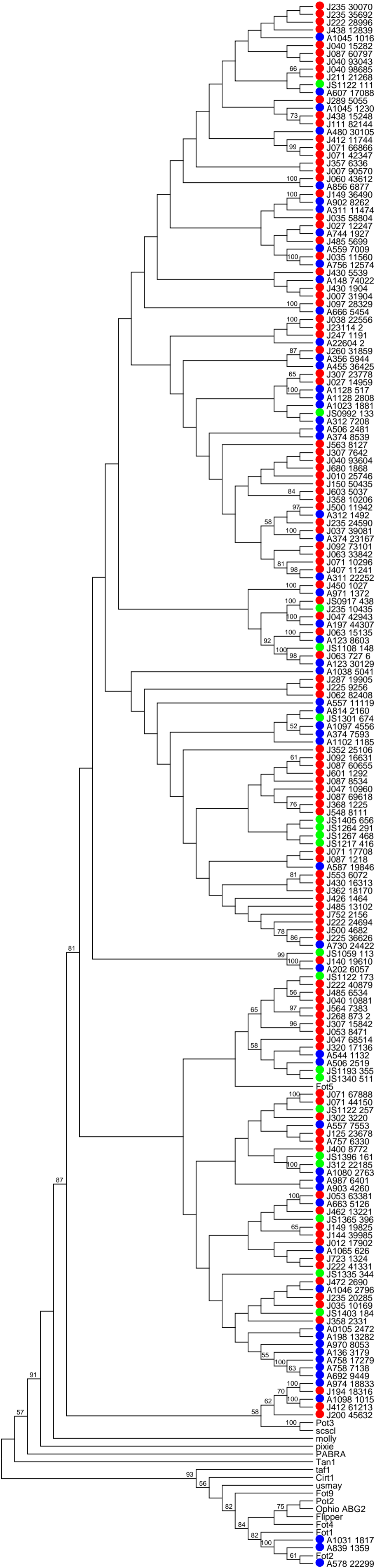

Supplement: Additional file 4: Figure S2. — Maximum likelihood tree of the Fot5 DDD catalytic domain. This analysis was done using the WAG substitution model [49] and gamma correction to account for among site rate variation. Percentage bootstrap support (based on a 1000 repeats, with cut-off value of 50 %) is indicated at the internodes. Genomic coordinates of putative Ceratocystis Fot5 homologs are provided in Additional file 3: Table S2. GenBank accession numbers or sequence identifiers for previously identified Fot5 homologs are: Fot2 [Genbank:JN624854, F. oxysporum), Fot5 [Genbank:CAE55867, F. oxysporum], Fot1 [Genbank:X64799, F. oxysporum], Fot4 [Genbank:AF076632, F. oxysporum], Fot9 [JGI:2517, F. graminearum], Fotyl [Genbank:CAG33729.1 Yarrowia lipolytica], Molly [Genbank:CAD32687, Parastagonospora nodorum], Ophio [Genbank:ABG26269, Ophiostoma novo-ulmi], PABRA [Genbank:ACY56713, Paracoccidioides brasiliensis], Pixie [Genbank:CAD32689, Parastagonospora nodorum], Pot2 [Genbank:CAA83918, Magnaporthe grisea], Pot3 [Genbank:AAC49418, M. grisea], SCSCL [Genbank:XP001592252, Sclerotinia sclerotiorum], Taf1 [Genbank:AAX83011, Aspergillus fumigatus], Tan1 [Genbank:U58946, Aspergillus awamori] USMA [Genbank:UM03882, Ustilago maydis), Flipper [Genbank:AAB63315, Botryotinia fuckeliana] and Cirt1 [Genbank:XP710204, Candida albicans]. For the Ceratocystis sequences JS = Ceratocystis albifundus (green dots), J = Ceratocystis manginecans (red dots) and A = Ceratocystis fimbriata (blue dots), followed by the genomic position. (PDF 59 kb) [file 12862_2015_550_MOESM4_ESM.pdf]
