# Supplementary material for: Saprophytic and pathogenic fungi in the Ceratocystidaceae differ in their ability to metabolize plant-derived sucrose
Source: BMC Evol Biol. 2015 Dec 7;15:273. doi: 10.1186/s12862-015-0550-7 (PMC4672557; doi:10.1186/s12862-015-0550-7)
Supplement: Additional file 3: Table S2. — List of the putative Fot5 homologs identified in this study. (DOC 160 kb) [file 12862_2015_550_MOESM3_ESM.doc]

**Additional file 3: Table S2 List of the putative Fot5 homologs identified in this study.**

| **Taxon^1^** | **Scaffold^2^** | |
| --- | --- | --- |
| *Ceratocystis fimbriata* | APWK02000105 | 2472 - 1779 |
| *Ceratocystis fimbriata* | APWK02000123 | 30129 - 31523 |
| *Ceratocystis fimbriata* | APWK02000123 | 8603 - 7812 |
| *Ceratocystis fimbriata* | APWK02000136 | 3179 - 4007 |
| *Ceratocystis fimbriata* | APWK02000148 | 74022 - 74840 |
| *Ceratocystis fimbriata* | APWK02000197 | 44307 - 45160 |
| *Ceratocystis fimbriata* | APWK02000198 | 13282 - 14288 |
| *Ceratocystis fimbriata* | APWK02000202 | 6057 - 4657 |
| *Ceratocystis fimbriata* | APWK02000311 | 11474 - 12699 |
| *Ceratocystis fimbriata* | APWK02000311 | 22252 - 23652 |
| *Ceratocystis fimbriata* | APWK02000312 | 1492 - 2416 |
| *Ceratocystis fimbriata* | APWK02000312 | 7208 - 6344 |
| *Ceratocystis fimbriata* | APWK02000356 | 5944 - 5183 |
| *Ceratocystis fimbriata* | APWK02000374 | 23167 - 22031 |
| *Ceratocystis fimbriata* | APWK02000374 | 7593 - 8337 |
| *Ceratocystis fimbriata* | APWK02000374 | 8539 - 9495 |
| *Ceratocystis fimbriata* | APWK02000455 | 36425 - 37405 |
| *Ceratocystis fimbriata* | APWK02000480 | 30105 - 28382 |
| *Ceratocystis fimbriata* | APWK02000506 | 2481 - 1694 |
| *Ceratocystis fimbriata* | APWK02000506 | 2519 - 3517 |
| *Ceratocystis fimbriata* | APWK02000544 | 1132 - 608 |
| *Ceratocystis fimbriata* | APWK02000557 | 11119 - 10841 |
| *Ceratocystis fimbriata* | APWK02000557 | 7553 - 6235 |
| *Ceratocystis fimbriata* | APWK02000559 | 7009 - 8409 |
| *Ceratocystis fimbriata* | APWK02000578 | 22299 - 22994 |
| *Ceratocystis fimbriata* | APWK02000587 | 19846 - 18485 |
| *Ceratocystis fimbriata* | APWK02000607 | 17088 - 17918 |
| *Ceratocystis fimbriata* | APWK02000663 | 5126 - 3661 |
| *Ceratocystis fimbriata* | APWK02000666 | 5454 - 4177 |
| *Ceratocystis fimbriata* | APWK02000692 | 9449 - 9747 |
| *Ceratocystis fimbriata* | APWK02000730 | 24422 - 23022 |
| *Ceratocystis fimbriata* | APWK02000744 | 1927 - 1367 |
| *Ceratocystis fimbriata* | APWK02000756 | 12574 - 13923 |
| *Ceratocystis fimbriata* | APWK02000757 | 6330 - 5743 |
| *Ceratocystis fimbriata* | APWK02000758 | 17279 |
| *Ceratocystis fimbriata* | APWK02000758 | 7138 - 6403 |
| *Ceratocystis fimbriata* | APWK02000814 | 2160 - 1530 |
| *Ceratocystis fimbriata* | APWK02000839 | 1359 - 147 |
| *Ceratocystis fimbriata* | APWK02000856 | 6877 - 5481 |
| *Ceratocystis fimbriata* | APWK02000902 | 8262 - 7294 |
| *Ceratocystis fimbriata* | APWK02000903 | 4260 - 5203 |
| *Ceratocystis fimbriata* | APWK02000970 | 8053 - 8768 |
| *Ceratocystis fimbriata* | APWK02000971 | 1372 - 230 |
| *Ceratocystis fimbriata* | APWK02000974 | 18833 - 20178 |
| *Ceratocystis fimbriata* | APWK02000987 | 6401 - 5892 |
| *Ceratocystis fimbriata* | APWK02001023 | 18815 - 18234 |
| *Ceratocystis fimbriata* | APWK02001031 | 18176 - 18733 |
| *Ceratocystis fimbriata* | APWK02001038 | 5041 - 3794 |
| *Ceratocystis fimbriata* | APWK02001045 | 10162 - 8913 |
| *Ceratocystis fimbriata* | APWK02001045 | 12306 - |
| *Ceratocystis fimbriata* | APWK02001046 | 2796 - 2038 |
| *Ceratocystis fimbriata* | APWK02001065 | 626 - 1698 |
| *Ceratocystis fimbriata* | APWK02001080 | 2763 - 4090 |
| *Ceratocystis fimbriata* | APWK02001097 | 4556 - 3542 |
| *Ceratocystis fimbriata* | APWK02001098 | 10156 - 11040 |
| *Ceratocystis fimbriata* | APWK02001102 | 11856 - 12170 |
| *Ceratocystis fimbriata* | APWK02001128 | 2808 - 2062 |
| *Ceratocystis fimbriata* | APWK02001128 | 517 - 1234 |
| *Ceratocystis fimbriata* | APWK02000925 and APWK02000924 | 22604 - 23811 |
| *Ceratocystis manginecans* | JJRZ01000038 | 36427-37962 |
| *Ceratocystis manginecans* | JJRZ01000007 | 31904 - 32740 |
| *Ceratocystis manginecans* | JJRZ01000007 | 90570 - 91646 |
| *Ceratocystis manginecans* | JJRZ01000010 | 25746 - 24799 |
| *Ceratocystis manginecans* | JJRZ01000012 | 179023 - 178172 |
| *Ceratocystis manginecans* | JJRZ01000027 | 122476 - 123591 |
| *Ceratocystis manginecans* | JJRZ01000027 | 149594 - 150571 |
| *Ceratocystis manginecans* | JJRZ01000035 | 101693 - 100920 |
| *Ceratocystis manginecans* | JJRZ01000035 | 115607 - 114546 |
| *Ceratocystis manginecans* | JJRZ01000035 | 58804 - 59439 |
| *Ceratocystis manginecans* | JJRZ01000037 | 39081 - 37951 |
| *Ceratocystis manginecans* | JJRZ01000038 | 22556 - 23808 |
| *Ceratocystis manginecans* | JJRZ01000040 | 108812 - 109645 |
| *Ceratocystis manginecans* | JJRZ01000040 | 15282 - 16682 |
| *Ceratocystis manginecans* | JJRZ01000040 | 93043 - 91826 |
| *Ceratocystis manginecans* | JJRZ01000040 | 93604 - 94776 |
| *Ceratocystis manginecans* | JJRZ01000040 | 98685 - 99658 |
| *Ceratocystis manginecans* | JJRZ01000047 | 109605 - 110621 |
| *Ceratocystis manginecans* | JJRZ01000047 | 42943 - 43647 |
| *Ceratocystis manginecans* | JJRZ01000047 | 68514 - 69785 |
| *Ceratocystis manginecans* | JJRZ01000053 | 63381 - 62626 |
| *Ceratocystis manginecans* | JJRZ01000053 | 8471 - 7500 |
| *Ceratocystis manginecans* | JJRZ01000060 | 43612 - 42422 |
| *Ceratocystis manginecans* | JJRZ01000062 | 82408 - 83190 |
| *Ceratocystis manginecans* | JJRZ01000063 | 15135 - 14359 |
| *Ceratocystis manginecans* | JJRZ01000063 | 33842 - 34771 |
| *Ceratocystis manginecans* | JJRZ01000063 | 727 - 68 |
| *Ceratocystis manginecans* | JJRZ01000071 | 102960 - 103565 |
| *Ceratocystis manginecans* | JJRZ01000071 | 17708 - 18418 |
| *Ceratocystis manginecans* | JJRZ01000071 | 42347 - 43342 |
| *Ceratocystis manginecans* | JJRZ01000071 | 44150 - 45061 |
| *Ceratocystis manginecans* | JJRZ01000071 | 66866 - 67495 |
| *Ceratocystis manginecans* | JJRZ01000071 | 67888 - 68775 |
| *Ceratocystis manginecans* | JJRZ01000087 | 1218 - 2234 |
| *Ceratocystis manginecans* | JJRZ01000087 | 60655 - 59912 |
| *Ceratocystis manginecans* | JJRZ01000087 | 60797 - 62197 |
| *Ceratocystis manginecans* | JJRZ01000087 | 69618 - 68743 |
| *Ceratocystis manginecans* | JJRZ01000087 | 8534 - 9682 |
| *Ceratocystis manginecans* | JJRZ01000092 | 16631 - 17467 |
| *Ceratocystis manginecans* | JJRZ01000092 | 73101 - 73814 |
| *Ceratocystis manginecans* | JJRZ01000097 | 28329 - 28961 |
| *Ceratocystis manginecans* | JJRZ01000111 | 82144 - 82884 |
| *Ceratocystis manginecans* | JJRZ01000125 | 23678 - 22878 |
| *Ceratocystis manginecans* | JJRZ01000140 | 19610 - 18201 |
| *Ceratocystis manginecans* | JJRZ01000144 | 39985 - 38576 |
| *Ceratocystis manginecans* | JJRZ01000149 | 19825 - 18758 |
| *Ceratocystis manginecans* | JJRZ01000149 | 36490 - 35312 |
| *Ceratocystis manginecans* | JJRZ01000150 | 50435 - 51702 |
| *Ceratocystis manginecans* | JJRZ01000194 | 18316 - 18996 |
| *Ceratocystis manginecans* | JJRZ01000200 | 45632 - 44712 |
| *Ceratocystis manginecans* | JJRZ01000211 | 21268 - 20648 |
| *Ceratocystis manginecans* | JJRZ01000222 | 24694 - 25881 |
| *Ceratocystis manginecans* | JJRZ01000222 | 28996 - 27719 |
| *Ceratocystis manginecans* | JJRZ01000222 | 40879 - 40034 |
| *Ceratocystis manginecans* | JJRZ01000222 | 41331 - 42740 |
| *Ceratocystis manginecans* | JJRZ01000225 | 36626 - 35349 |
| *Ceratocystis manginecans* | JJRZ01000225 | 9256 - 8657 |
| *Ceratocystis manginecans* | JJRZ01000235 | 10435 - 9374 |
| *Ceratocystis manginecans* | JJRZ01000235 | 20285 - 21352 |
| *Ceratocystis manginecans* | JJRZ01000235 | 24590 - 23337 |
| *Ceratocystis manginecans* | JJRZ01000235 | 30070 - 31080 |
| *Ceratocystis manginecans* | JJRZ01000235 | 35692 - 34808 |
| *Ceratocystis manginecans* | JJRZ01000247 | 1191 - 265 |
| *Ceratocystis manginecans* | JJRZ01000260 | 31859 - 32488 |
| *Ceratocystis manginecans* | JJRZ01000268 | 873 - 235 |
| *Ceratocystis manginecans* | JJRZ01000287 | 19905 - 19222 |
| *Ceratocystis manginecans* | JJRZ01000289 | 5055 - 3796 |
| *Ceratocystis manginecans* | JJRZ01000302 | 3220 - 2561 |
| *Ceratocystis manginecans* | JJRZ01000307 | 15842 - 16795 |
| *Ceratocystis manginecans* | JJRZ01000307 | 23778 - 22906 |
| *Ceratocystis manginecans* | JJRZ01000307 | 7642 - 8640 |
| *Ceratocystis manginecans* | JJRZ01000312 | 22185 - 21553 |
| *Ceratocystis manginecans* | JJRZ01000320 | 17136 - 16378 |
| *Ceratocystis manginecans* | JJRZ01000352 | 25106 - 24237 |
| *Ceratocystis manginecans* | JJRZ01000357 | 6336 - 7217 |
| *Ceratocystis manginecans* | JJRZ01000358 | 10206 - 9196 |
| *Ceratocystis manginecans* | JJRZ01000358 | 2331 - 3602 |
| *Ceratocystis manginecans* | JJRZ01000362 | 18170 - 16683 |
| *Ceratocystis manginecans* | JJRZ01000368 | 1225 - 1839 |
| *Ceratocystis manginecans* | JJRZ01000400 | 8772 - 7999 |
| *Ceratocystis manginecans* | JJRZ01000407 | 11241 - 9970 |
| *Ceratocystis manginecans* | JJRZ01000412 | 11744 - 12691 |
| *Ceratocystis manginecans* | JJRZ01000412 | 61213 - 61836 |
| *Ceratocystis manginecans* | JJRZ01000426 | 1464 - 193 |
| *Ceratocystis manginecans* | JJRZ01000430 | 16313 - 17260 |
| *Ceratocystis manginecans* | JJRZ01000430 | 1904 - 1305 |
| *Ceratocystis manginecans* | JJRZ01000430 | 5539 - 6321 |
| *Ceratocystis manginecans* | JJRZ01000438 | 12839 - 11976 |
| *Ceratocystis manginecans* | JJRZ01000438 | 15248 - 15988 |
| *Ceratocystis manginecans* | JJRZ01000450 | 1027 - 2178 |
| *Ceratocystis manginecans* | JJRZ01000462 | 13221 - 14288 |
| *Ceratocystis manginecans* | JJRZ01000472 | 2690 - 3682 |
| *Ceratocystis manginecans* | JJRZ01000485 | 13102 - 11831 |
| *Ceratocystis manginecans* | JJRZ01000485 | 5699 - 4422 |
| *Ceratocystis manginecans* | JJRZ01000485 | 6534 - 7811 |
| *Ceratocystis manginecans* | JJRZ01000500 | 11942 - 12619 |
| *Ceratocystis manginecans* | JJRZ01000500 | 4682 - 3405 |
| *Ceratocystis manginecans* | JJRZ01000548 | 8111 - 7236 |
| *Ceratocystis manginecans* | JJRZ01000553 | 6072 - 7223 |
| *Ceratocystis manginecans* | JJRZ01000563 | 8127 - 7312 |
| *Ceratocystis manginecans* | JJRZ01000564 | 7383 - 6745 |
| *Ceratocystis manginecans* | JJRZ01000601 | 1292 - 2569 |
| *Ceratocystis manginecans* | JJRZ01000603 | 5037 - 6227 |
| *Ceratocystis manginecans* | JJRZ01000680 | 1868 - 597 |
| *Ceratocystis manginecans* | JJRZ01000723 | 1324 - 71 |
| *Ceratocystis manginecans* | JJRZ01000752 | 2156 - 2938 |
| *Ceratocystis albifundus* | JSSU01000917 | 4384 - 3785 |
| *Ceratocystis albifundus* | JSSU01000992 | 13389 - 13150 |
| *Ceratocystis albifundus* | JSSU01001059 | 11392 - 11984 |
| *Ceratocystis albifundus* | JSSU01001108 | 14800 - 14189 |
| *Ceratocystis albifundus* | JSSU01001122 | 11103 - 10804 |
| *Ceratocystis albifundus* | JSSU01001122 | 17389 - 17033 |
| *Ceratocystis albifundus* | JSSU01001122 | 25755 - 25381 |
| *Ceratocystis albifundus* | JSSU01001193 | 35553 - 35521 |
| *Ceratocystis albifundus* | JSSU01001217 | 41643 - 42286 |
| *Ceratocystis albifundus* | JSSU01001264 | 29167 - 28544 |
| *Ceratocystis albifundus* | JSSU01001267 | 46882 - 46499 |
| *Ceratocystis albifundus* | JSSU01001301 | 6742 - 7030 |
| *Ceratocystis albifundus* | JSSU01001335 | 34450 - 34863 |
| *Ceratocystis albifundus* | JSSU01001340 | 51154 - 50897 |
| *Ceratocystis albifundus* | JSSU01001365 | 3963 - 3352 |
| *Ceratocystis albifundus* | JSSU01001396 | 161193 - 161116 |
| *Ceratocystis albifundus* | JSSU01001403 | 184780 - 184155 |
| *Ceratocystis albifundus* | JSSU01001405 | 65665 - 65318 |

^1^ The GenBank accesssion numbers for the *C. manginecans*, *C. fimbriata* and *C. albifundus* genomes are JJRZ01000000, APWK00000000 and JSSU00000000.

^2^ The name of the contig is followed by the nucleotide position of the gene within the contig.
